# The modifying effect of mutant *LRRK2* on mutant *GBA1*-associated Parkinson disease

**DOI:** 10.1093/hmg/ddaf062

**Published:** 2025-05-02

**Authors:** Vera Serebryany-Piavsky, Lian Egulsky, Julia Elia Manoim-Wolkovitz, Saar Anis, Sharon Hassin-Baer, Moshe Parnas, Mia Horowitz

**Affiliations:** Shmunis School of Biomedicine and Cancer Research, Tel-Aviv University, Levanon St., Tel Aviv 69978, Israel; Shmunis School of Biomedicine and Cancer Research, Tel-Aviv University, Levanon St., Tel Aviv 69978, Israel; Department of Physiology and Pharmacology, Faculty of Medicine, Tel Aviv University, Levanon St., Tel Aviv 69978, Israel; Movement Disorders Institute, Department of Neurology, Sheba Medical Center, Tel-Hashomer, Ramat-Gan 52620, Israel; Movement Disorders Institute, Department of Neurology, Sheba Medical Center, Tel-Hashomer, Ramat-Gan 52620, Israel; Department of Physiology and Pharmacology, Faculty of Medicine, Tel Aviv University, Levanon St., Tel Aviv 69978, Israel; Sagol School of Neuroscience, Tel Aviv University, Levanon St., Tel Aviv 69978, Israel; Shmunis School of Biomedicine and Cancer Research, Tel-Aviv University, Levanon St., Tel Aviv 69978, Israel; Sagol School of Neuroscience, Tel Aviv University, Levanon St., Tel Aviv 69978, Israel

**Keywords:** *GBA1*, *LRRK2*, Parkinson disease, *Drosophila*

## Abstract

Parkinson disease (PD) is the second most common neurodegenerative disease. While most cases are sporadic, in ~ 5%–10% of PD patients the disease is caused by mutations in several genes, among them *GBA1* (glucocerebrosidase beta 1) and *LRRK2* (leucine-rich repeat kinase 2), both prevalent among the Ashkenazi Jewish population. *LRRK2-*associated PD tends to be milder than *GBA1*-associated PD. Several recent clinical studies have suggested that carriers of both *GBA1* and *LRRK2* mutations develop milder PD compared to that observed among *GBA1* carriers. These findings strongly suggested an interplay between the two genes in the development and progression of PD. In the present study *Drosophila* was employed as a model to investigate the impact of mutations in the *LRRK2* gene on mutant *GBA1*-associated PD. Our results strongly indicated that flies expressing both mutant genes exhibited milder parkinsonian signs compared to the disease developed in flies expressing only a *GBA1* mutation. This was corroborated by a decrease in the ER stress response, increase in the number of dopaminergic cells, elevated levels of tyrosine hydroxylase, reduced neuroinflammation, improved locomotion and extended survival. Furthermore, a significant decrease in the steady-state levels of mutant *GBA1*-encoded GCase was observed in the presence of mutant *LRRK2*, strongly implying a role for mutant LRRK2 in degradation of mutant GCase.

## Introduction

Parkinson disease (PD) is a common neurodegenerative disorder, caused by multifactorial etiology involving genetic susceptibility, exposure to environmental factors and normal aging processes [1]. The neurodegenerative features of PD are characterized by progressive degeneration of dopaminergic neurons and pathological accumulation of α-synuclein in cells of the central and peripheral nervous systems [2, 3]. These changes induce both motor symptoms and a broad range of non-motor symptoms [4]. The degeneration of dopaminergic neurons in the substantia nigra pars compacta causes PD motor symptoms such as resting tremor, bradykinesia, and postural instability [5]. Common non-motor symptoms include psychiatric and mood disorders [6], constipation [7], excessive somnolence [8], erectile dysfunction [9] and olfactory loss [10]—some of which may emerge 5–20 years prior to motor symptoms onset, during the prodromal stage of the disease [11, 12]. Neuroinflammation is also a prominent feature of PD pathology [13]. Although most PD cases are sporadic, approximately 5%–10% result from mutations in a growing number of genes [14], although with considerable variability in their risk effects between populations. The two most common mutated genes associated with familial PD are *GBA1* and *LRRK2* [15–17]. *LRRK2* variations account for approximately 2% of all PD cases globally, but are significantly higher in specific ethnic groups, particularly among individuals of Ashkenazi Jewish (G2019S mutation, 28%) or Arabian Berber (G2019S mutation, 36%) descents [18]. Penetrance of *LRRK2* G2019S in Ashkenazi Jews is only 26% [19]. Risk variants in *GBA1* are found in up to 10% of PD cases worldwide and in ~ 7%–20% of PD cases among individuals of Ashkenazi Jewish descent [20]. Penetrance of *GBA1* variants ranges from 9% to 19% depending on age [21] and severity of mutation [22].

Biallelic mutations in the *GBA1* gene cause Gaucher disease (GD), with high carrier prevalence (1:15) among Ashkenazi Jews [23, 24]. *GBA1* encodes lysosomal acid β-glucocerebrosidase (GCase), which degrades lysosomal glucosylceramide (GlcCer) [25–27], therefore, decreased lysosomal activity leads to GlcCer accumulation. Diacylation of GlcCer by lysosomal acid ceramidase leads to the accumulation of glucosylsphingosine [28–30]. Due to its heterogeneity, GD is divided into non-neuronopathic and neuronopathic forms. Type 1 GD (GD1), most prevalent among Ashkenazi Jews, is usually not associated with neurological symptoms. Type 2 (GD2) and Type 3 (GD3) are the neuronopathic forms of GD [26, 27]. The most prevalent mutation among GD1 is the N370S mutation (classical nomenclature; N409S, according to the new HUGO nomenclature) [31]. The pan ethnic L444P mutation (classical nomenclature; L483P, according to the new HUGO nomenclature) is associated, in homozygosity, with GD3 [32].

Carriers of monoallelic *GBA1* mutations and GD1 patients have a higher propensity to develop PD in comparison to the non-mutant *GBA1* associated population, indicating that a mutant *GBA1* (*mGBA1*) allele is a predisposing factor for development of PD [20, 33, 34]. *GBA1*-associated PD is characterized by an earlier onset, and more severe cognitive and non-motor symptoms in comparison to sporadic cases [20, 35, 36]. While most carriers of *GBA1* mutations do not develop PD, severe *GBA1* mutations (like L444P) result in a higher risk of PD and a faster progressing disease course than PD resulting from milder mutations (like N370S) [36, 37].

GCase is synthesized on endoplasmic reticulum (ER) bound polyribosomes and, following proper folding in the ER, is transported to the lysosomes. Mutations in *GBA1* result in misfolded GCase that is retained in the ER by the quality control machinery designed to facilitate proper folding [38, 39]. This retention causes ER stress, which upregulates the ER stress response, known as the Unfolded Protein Response (UPR) [40, 41]. An integral part of the UPR is ER Associated Degradation (ERAD), which targets misfolded proteins in the ER for cytoplasmic ubiquitination and subsequent proteasomal degradation [42]. In previous studies, we provided evidence that the transgenic overexpression of human misfolded mutant GCase variants in the flies’ dopaminergic cells, leads to development of a parkinsonian-like phenotype [43, 44], ultimately contributing to age dependent death of dopaminergic cells, motor deterioration and decreased survival. Similar results were documented by Sanchez et al [45]. Additionally, transgenic expression of human mutant GCase (mGCase) in eye photoreceptors caused neurodevelopmental and morphological abnormalities [46]. Overall, the findings demonstrated that the severity of parkinsonian symptoms correlated with the severity of the human mutation expressed in the fly. These studies collectively highlighted a 'gain-of-function' mechanism associated with mutant human *GBA1* variants overexpressed in the fly model. Nevertheless, it should be noted that a recent publication [47] has shown the existence of *GBA1* transcripts, mainly in the brain, which encode non-lysosomal *GBA1* proteins with no GCase activity. These novel *GBA1* isoforms may contribute to phenotypic diversity in GD and PD.

The *LRRK2* gene encodes a large multi-domain protein, with both kinase and GTPase activities [48]. It plays a multifaceted role in numerous cellular processes, including protein sorting, cytoskeletal dynamics, vesicular trafficking and neurotransmitter release, mitochondrial homeostasis, macroautophagy, chaperone mediated autophagy and lysosomal positioning, Golgi and retromer positioning and protein synthesis [49–51]. Despite the vast literature on the intracellular activities of LRRK2, the majority of studies point to disrupted vesicle trafficking as a central alteration [52]. Most pathogenic mutations in the *LRRK2* gene increase its kinase activity or reduce its GTPase activity [53]. The dominant inheritance of *LRRK2*-associated PD suggests a ‘gain-of-function’ mechanism [54]. The G2019S mutation, prevalent among Ashkenazi Jews, is localized within the kinase domain of *LRRK2* and enhances V_max_ for kinase activity [55]. The I2020T mutation is considered similar in effect to the G2019S mutation [50, 56]. Both mutations cause hyperphosphorylation of several Rab GTPase proteins, leading to abnormal intracellular trafficking events [57–59].

In recent years, several publications documented an interplay between *GBA1*-encoded mGCase and mutant LRRK2 (mLRRK2). Some publications suggested that mLRRK2 acts as a negative regulator of GCase activity. One such research used iPSCs prepared from fibroblasts of PD patients with *LRRK2* mutations, that were differentiated to dopaminergic cells. When LRRK2 kinase activity was inhibited in these cells, an increase in GCase activity was noted [60]. Another publication demonstrated morphological and functional lysosomal damage in both heterozygous and homozygous *GBA1* D409V knock-in mouse astrocytes and in human iPSC-derived neurons with CRISPR/Cas9 edited allelic heterozygous loss of *GBA1* [61]. Abnormalities were normalized by LRRK2 kinase inhibition.

Results of other publications implied a possible protective effect of mLRRK2 on mGCase. Clinical studies by Yahalom et al. [62] showed that while *GBA1*-PD was characterized by higher rates of non-motor parkinsonian symptoms, it seemed that these features were somewhat rescued in patients who carried mutations in both their *GBA1* and *LRRK2* genes. These results were confirmed by other publications [63, 64]. In addition, it has been noted that PD patients who carried a G2019S *LRRK2* mutation had higher GCase activity than non-carriers with concomitant elevation in levels of the lysosomal enzymes acid sphingomyelinase and alpha galactosidase A [65, 66]. This result strongly suggested that the presence of a mutant *LRRK2* enhances intracellular trafficking of these enzymes, thus leading to an increase in their lysosomal activity.

We decided to test a possible interplay between m*GBA1* and *mLRRK2* in the fly *Drosophila melanogaster*. *Drosophila* serves as a convenient animal model in which genetic interaction between two genes or the gene products, and not necessarily physical interaction between the gene products, can be studied [67, 68].

For expression of human genes in the fly the UAS (upstream activating sequences)-GAL4 system has been employed. This bipartite system consists of a GAL4 driver line, which expresses the yeast transcription activator protein GAL4 under the control of a native fly promoter, and a UAS responder line, where the gene of interest is coupled to a UAS, to which GAL4 specifically binds [69]. When the two lines are crossed, the progeny inherit both components, enabling expression of GAL4 in tissues where the driver is active. GAL4 binding to the UAS element activates transcription, resulting in targeted gene expression. Importantly, the UAS-GAL4 system results in gene overexpression [70].

In the present study we tested a possible interplay between m*GBA1* and m*LRRK2* in *Drosophila*. To this end, we overexpressed human m*GBA1* variants and human m*LRRK2* in the fly to provide support to the observations that carriers of both human *GBA1* and *LRRK2* mutations develop a milder form of PD compared to that manifested in carriers of *GBA1* mutations. These findings provide compelling evidence of a genetic interplay between *GBA1* and *LRRK2* in PD, highlighting the potential therapeutic implications of targeting this interaction to modulate disease progression.

## Results

### GCase activity and glucosylceramide levels in transgenic flies

**Figure 1:**
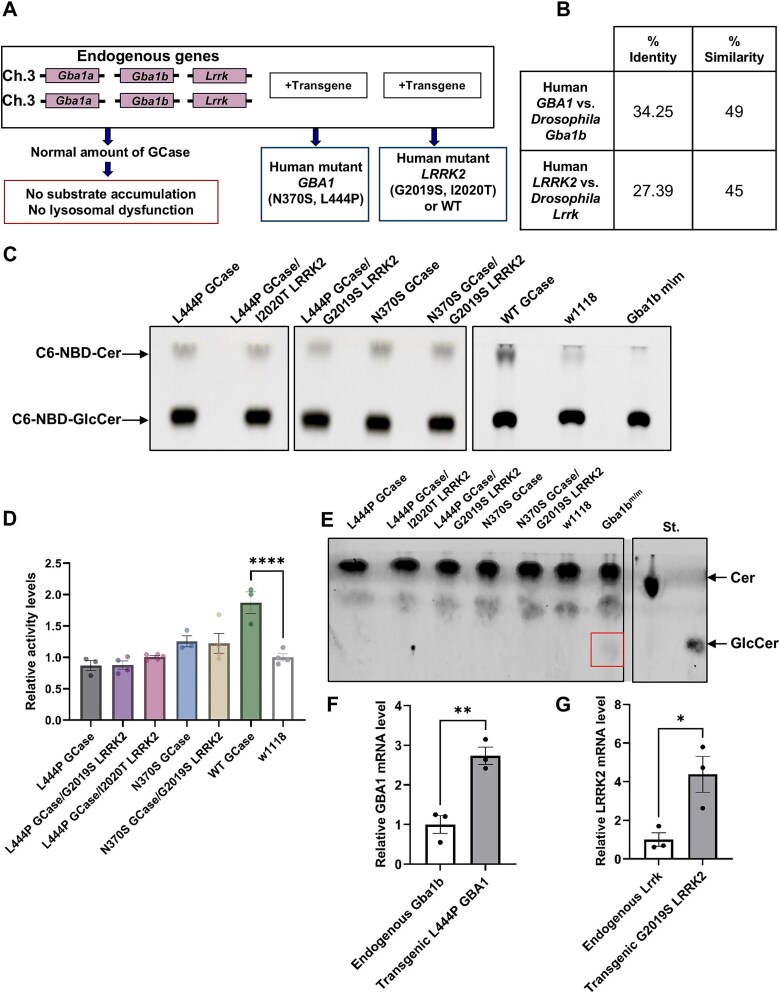
GCase activity and GlcCer (glucosylceramide) in transgenic flies. (A) Schematic representation of the regions on fly chromosome 3, which contains the two *GBA1* orthologs (*Gba1a*, *Gba1b*) and the *LRRK2* ortholog (*LRRK*). The transgenes are either: Mutant human *GBA1*, mutant human *LRRK2* or both together. The human *GBA1* variants include: N370S, L444P. The human *LRRK2* variants include: I2020T, G2019S. WT *LRRK2* expressing flies were also used. (B) a table summarizing amino acid identity and similarity of *GBA1* and *LRRK2* between human and *drosophila* (UniProt). (C) a TLC plate presenting activity assay of GCase as measured in lysates prepared from 2-days old flies. The value obtained from *Gba1b^m/m^* flies was considered as background [71] and subtracted from all other samples. (D) Average GCase activity of flies expressing L444P GCase (grey bars), L444P GCase and G2019S LRRK2 (purple bars), L444P GCase and I2020T LRRK2 (pink bars), N370S GCase (blue bars), N370S GCase and G2019S LRRK2 (brown bars), WT GCase (green bars) and w1118 (clear bars). The results are the mean ± SEM of three independent experiments. One-way ANOVA was used to calculate the significance of the results (F [6, 22] = 4.152, *P* = 0.0061). (E) TLC plate showing no GlcCer accumulation in 12-days old flies in comparison to its accumulation in *Gba1b^m/m^* flies. St.—Standards. Expression was under a Da-GAL4 driver. (F) mRNA level of *GBA1* (grey bar) and endogenous fly *Gba1b* (clear bar) were evaluated using quantitative RT-PCR analysis in flies expressing mutant human *GBA1*. The results are the mean ± SEM of three independent experiments. Paired student’s t-test was used to calculate the significance of the results (p = 0.0052). (G) mRNA level of *LRRK2* (grey bar) and endogenous fly *LRRK* (clear bar) were evaluated using quantitative RT-PCR analysis in flies expressing mutant human *LRRK2*. The results are the mean ± SEM of three independent experiments. Paired student’s t-test was used to calculate the significance of the results (*P* = 0.0274).

In the present study we aimed to test the interplay between human m*GBA1*-encoded GCase and human m*LRRK2* variants, expressed in the fly. The *Drosophila* genome contains two *GBA1* orthologs (*Gba1a* and *Gba1b*, of which only *Gba1b* is a *bona fide* GCase encoding gene [71]) as well as a single *LRRK2* ortholog (Fig. 1A and B). Thus, it was important to validate that flies expressing the different human *GBA1* variants do not display abnormal levels of GCase activity or substrate accumulation, which could potentially affect the results. GCase activity was tested in lysates prepared from flies expressing human L444P or N370S *GBA1* variants or from double mutant flies expressing L444P or N370S *GBA1* in combination with G2019S or I2020T *LRRK2* variants, using the artificial substrate C6-NBD-GlcCer [72].

The results indicated that flies expressing the wild type (WT) human GCase exhibited approximately double the activity of w^1118^ flies while flies expressing either the human N370S or the L444P *GBA1* variants were not statistically different than the control w^1118^ flies, consistent with previous results [73] (Fig. 1C and D). Likewise, GCase activity in double mutant flies (i.e. mutants in both *GBA1* and *LRRK2*) was similar to that of flies expressing only a mutant *GBA1* and w^1118^ flies (Fig. 1C and D).

We further tested possible GlcCer accumulation in the flies, using Thin Layer Chromatography (TLC). No detectable substrate accumulation was observed in the tested lines in comparison to *Gba1b*^m/m^ flies, which have a deletion in their endogenous *GBA1b* gene and serve as a positive control for GlcCer accumulation [71] (Fig. 1E). We also compared RNA levels of the expressed *GBA1* and *LRRK2* transgenes to the endogenous fly genes. The results indicated a ~ 3–4-fold elevation in expression under the *dopa decarboxylase* (Ddc)-GAL4 driver compared to the endogenous expression of the same genes (Fig. 1F and G). Importantly, the expression level of the different transgenes under the Ddc-GAL4 driver was comparable (Fig. S1A and B). Taken together, the presented results demonstrate that flies expressing a mutant *GBA1* variant or a double mutant *GBA1* and a *LRRK2* variants do not exhibit any gross change in GCase activity nor in its substrate accumulation.

### Expression of mutant LRRK2 decreases mutant *GBA1*-associated UPR

**Figure 2:**
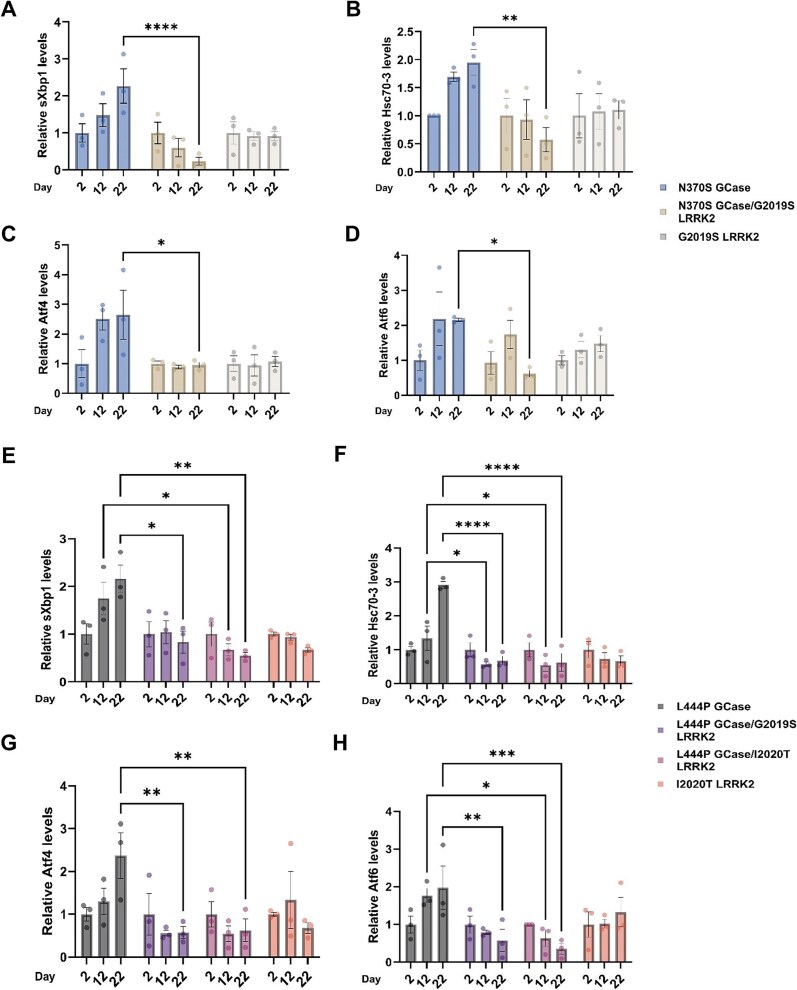
UPR parameters are decreased in flies co-expressing mGBA1 and mLRRK2. mRNA levels of UPR markers (a,E) sXbp1, (B,F) Hsc-70-3, (C,G) Atf4 and (D,H) Atf6 in whole flies at days 2, 12 and 22 post-eclosion, expressing N370S GCase (blue bars), N370S GCase and G2019S LRRK2 (brown bars), G2019S LRRK2 (white bars), as well as L444P GCase (grey bars), L444P GCase and G2019S LRRK2 (purple bars), L444P GCase and I2020T LRRK2 (pink bars), I2020T LRRK2 (orange bars), were analyzed by qRT-PCR. Expression was under a Da-GAL4 driver. The results are the mean ± SEM of three independent experiments. Two-way ANOVA was used to calculate significance of the results: * < 0.05, ** < 0.01, *** < 0.001, **** < 0.0001.

As mentioned above, a fraction of mutant misfolded GCase is retained in the ER [38]. This chronic retention of mutant GCase in the ER leads to ER stress, which activates the UPR [43, 44, 46]. Since we have previously demonstrated that transgenic flies expressing *mGBA1* alleles exhibit activation of the UPR and develop parkinsonian-like phenotypes [43, 44], we opted to use the same model to investigate the impact of m*LRRK2* transgenes on *GBA1*-associated phenotypes. Specifically, we tested the effect of G2019S *LRRK2* on N370S *GBA1*, a combination observed in Ashkenazi Jewish PD patients [62–64] and on L444P *GBA*1 variant, previously reported in a Brazilian family [54]. We also tested the effect of I2020T *LRRK2* on L444P *GBA1* variant*,* a hypothetical combination, which has not been documented among PD patients. We measured UPR activation by elevation in mRNAs of the BiP ortholog heat-shock cognate 70–3 (*Hsc-70-3* gene) [74], and in the levels of *Atf4* [75] and *Atf6* [76], using qRT-PCR with primers specific for the *Drosophila* genes. We also tested the mRNA levels of spliced *Xbp1* [77], as illustrated in Fig. S2. Given the age-related decline in cellular capacity to manage protein folding [78] and since we have previously shown that UPR parameters increase in flies expressing mutant GCase variants in an age dependent manner, we assessed UPR parameters at three different ages. A significant decrease in the expression level of the tested markers in 22-days old flies expressing mGCase and mLRRK2 was observed in comparison to flies expressing only mGCase (Fig. 2A–H). In the case of the more severe L444P mutation, differences in UPR levels were already evident in 12-days old flies (Fig. 2E–H).

**Figure 3:**
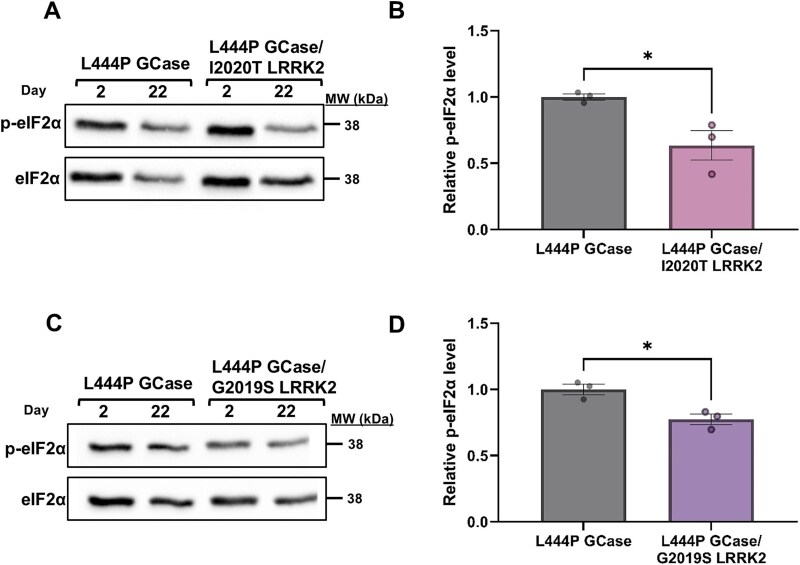
Phosphorylation of eIF2α is decreased in flies co-expressing m*GBA1* and m*LRRK2*. A protein lysates, prepared from 10 flies at days 2 and 22 post-eclosion, expressing either L444P GCase (grey bars) or L444P GCase and I2020T LRRK2 (pink bars), were subjected to SDS-PAGE and western blotting. Expression was under a Da-GAL4 driver. The corresponding blots were interacted with anti-phospho-eIF2α antibodies. As a loading control, the blots were interacted with anti-eIF2α antibodies. B intensities of the corresponding bands as shown in (A) were quantified by densitometry and the p-eIF2α amount was divided by that of eIF2α at the same lane. The value obtained for 22-days old double mutant flies was considered 1 (paired student’s t-test, p = 0.0324). The results are the mean ± SEM of three independent experiments. C same as (A), using flies expressing either L444P GCase (grey bars) or L444P GCase and G2019S LRRK2 (purple bars). D intensities of the corresponding bands as shown in (C) were quantified by densitometry and the amount of p-eIF2α was divided by that of eIF2α at the same lane. The value obtained for 22-days old double mutant flies was considered 1 (paired student’s t-test, p = 0.0159). The results are the mean ± SEM of three independent experiments.

UPR induces PERK activity, a kinase that mediates phosphorylation of the eukaryotic translation initiation factor 2α (eIF2α). Phosphorylation of eIF2α inhibits GDP/GTP exchange reaction, thus causing attenuation of ER-bound protein translation [79]. We tested possible changes in the phosphorylation level of eIF2α (p-eIF2α), using western blotting and interaction with anti-phosphorylated eIF2α antibodies. The results showed a significant decrease in levels of p-eIF2α at day 22 post-eclosion in L444P *GBA1*/I2020T *LRRK2* and L444P *GBA1*/G2019S *LRRK2* flies compared to that in L444P *GBA1* flies (Fig. 3A–D). Flies expressing only *mLRRK2* did not present elevation of any of the tested UPR markers at the tested ages.

Altogether, the results indicate that the presence of mLRRK2 lowers mGCase-associated UPR.

### Parkinsonian signs in flies co-expressing m*GBA1* and m*LRRK2* variants are less severe than in flies expressing only m*GBA1* variant

**Figure 4:**
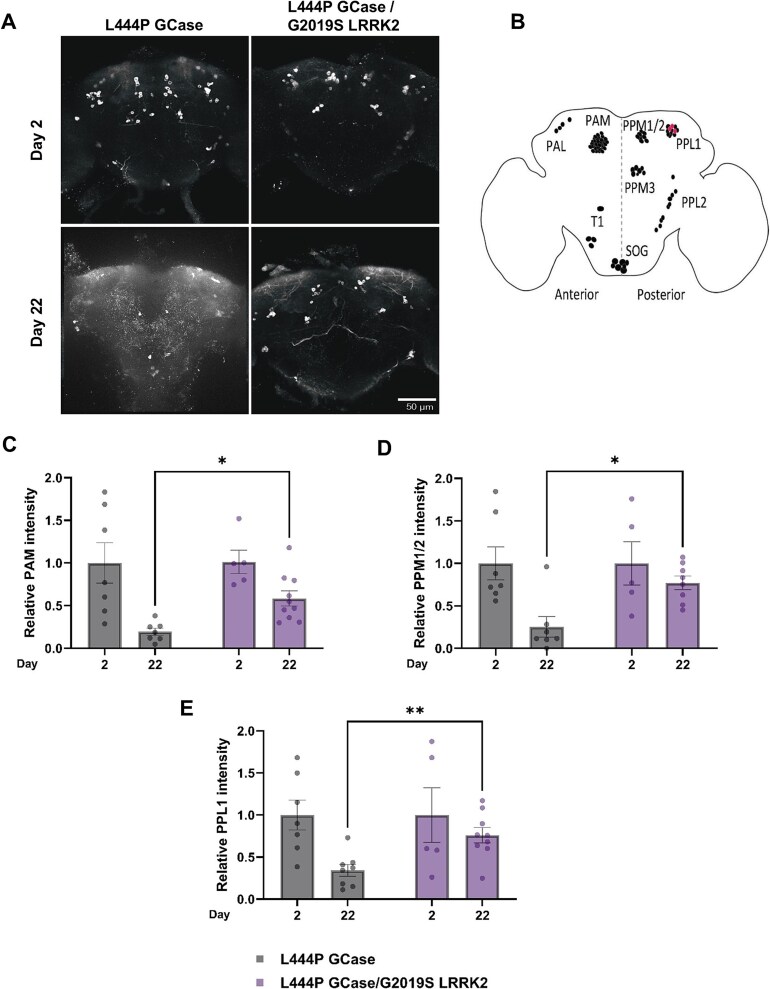
Degeneration of dopaminergic neurons is ameliorated in flies co-expressing m*GBA1* and m*LRRK2.* (A) Maximum intensity projection of 175 102;172 139 (left to right; top to bottom, respectively, 0.5 μm), confocal sections through the central brain of adult *drosophila* carrying L444P GCase and of flies carrying both L444P GCase and G2019S LRRK2 mutations, following their staining with anti-TH antibodies, at the ages of 2- and 22-days post-eclosion. (B) Schematic representation of dopaminergic neuronal clusters in the adult *drosophila* brain, according to Casture et al. [125] (C-E) quantification of the anti-TH signal intensity obtained from the brains described above (A), of flies carrying L444P GCase (grey bars) and of flies carrying both L444P GCase and G2019S LRRK2 mutations (purple bars). The results represent the mean ± SEM of 5–10 brains, in the clusters: Protocerebral anterior median (PAM, C), protocerebralmedial 1/2 (PPM1/2, D), and protocerebralanterior lateral 1 (PPL1, E). The clusters: Tritocerebral (T) and subesophageal (SOG) were not included. Paired student’s t-test, with Bonferroni-Dunn correction for multiple comparisons, was used to calculate the significance of the results. Significance: * < 0.05, ** < 0.01.

**Figure 5:**
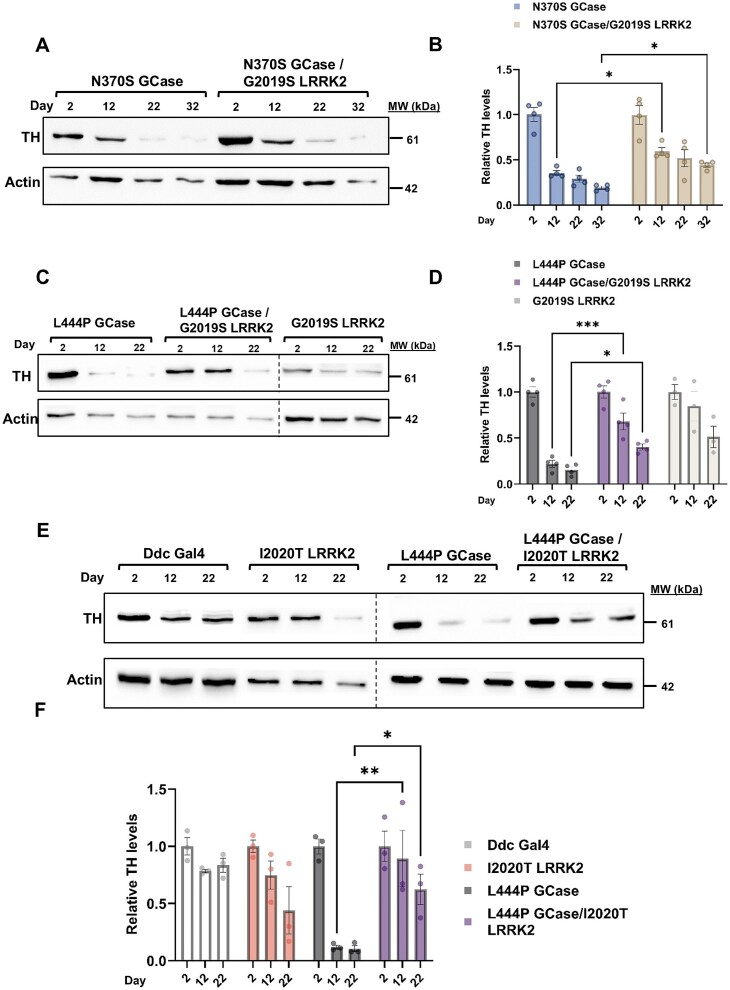
TH levels are higher in flies co-expressing m*GBA1* and m*LRRK2.* (A) Protein lysates were prepared from heads of 10 flies at days 2, 12 and 22 post-eclosion, expressing either N370S *GBA1* (blue bars) or N370S *GBA1* and G2019S *LRRK2* (brown bars), and subjected to western blotting. The corresponding blots were interacted with anti-TH antibodies. As a loading control, the blots were interacted with anti-actin antibody. Expression was under a Ddc-GAL4 driver. (B) Intensities of the corresponding bands as presented in (A) were quantified by densitometry and the TH amount was divided by that of actin at the same lane. The value obtained for 2-days old flies for each genotype was considered 1. The results are the mean ± SEM of four independent experiments. Two-way ANOVA analysis was used to calculate the significance of the results (day 12 p = 0.0455, day 22 p = 0.0332). (C) the same as (A), using flies expressing either L444P *GBA1* (grey bars), G2019S *LRRK2* (white bars) or both L444P *GBA1* and G2019S *LRRK2* (purple bars)*.* (D) Intensities of the corresponding bands as presented in (C) were quantified by densitometry and the TH amount was divided by that of actin at the same lane. The value obtained for 2-days old flies for each genotype was considered 1. The results are the mean ± SEM of four independent experiments. Two-way ANOVA analysis was used to calculate the significance of the results (day 12 p < 0.001, day 22 p = 0.04)*.* (E) the same as (A), using flies expressing Ddc-Gal4 (clear bars), I2020T *LRRK2* (pink bars), and either L444P *GBA1* (grey bars) or L444P *GBA1* and I2020T *LRRK2* (purple bars)*.* (F) Intensities of the corresponding bands as presented in (E) were quantified by densitometry and the TH amount was divided by that of actin at the same lane. The value obtained for 2-days old flies for each genotype was considered 1. The results are the mean ± SEM of three independent experiments. Two-way ANOVA analysis was used to calculate the significance of the results (day 12 p = 0.0011, day 22 p = 0.0466).

We and others have shown in the past that flies, expressing m*GBA1* variants in their dopaminergic cells, develop known human parkinsonian signs, including death of these cells, neuroinflammation, motor disabilities and shorter life span [43, 44, 46]. All mentioned parameters were tested in flies expressing a m*GBA1* variant (N370S or L444P) in comparison to flies expressing m*GBA1* and m*LRRK2* (I2020T or G2019S) variants together. To follow dopaminergic cell death, adult fly brains were stained with anti-Tyrosine Hydroxylase (TH) antibodies, in which expression was under a Ddc-GAL4 driver, and examined using confocal microscopy (Fig. 4A–E, Fig. S3A–D). In the *Drosophila* brain, dopaminergic neurons are organized in bilaterally symmetrical clusters (Fig. 4B). We quantified fluorescence intensity from three major dopaminergic neuron clusters: the protocerebral anterior medial (PAM) (Fig. 3C), the protocerebral posterior medial (PPM1/2) (Fig. 4D) and the posterior protocerebrum lateral (PPL1) (Fig. 4E). Flies expressing m*GBA1* alone exhibited a significantly greater reduction of the fluorescence signal at day 22 post eclosion, compared to flies expressing m*GBA1* and m*LRRK2* variants together, indicating enhanced dopaminergic neuron survival in the double mutant flies. Death of dopaminergic cells was further tested by measuring TH levels in heads of adult flies, using western blotting (Fig. 5). Double mutant flies displayed a significant increase in the amount of TH compared to flies expressing only mGCase (Fig. 5A–D). As expected, damage to dopaminergic cells in the N370S GCase expressing flies was less severe than that caused by the L444P GCase, (compare Fig. 5A and B and Fig. 5C and D). Interestingly, the decrease in TH level in flies expressing a m*LRRK2* variant was significantly milder than that in flies expressing a m*GBA1* variant (Fig. 5C–F). There was no significant difference between the tested *LRRK2* variants (G2019S versus I2020T) regarding the improvement in TH levels observed in the double mutant flies (two-way ANOVA: Day 12, p = 0.54; Day 22, *P* = 0.51). Altogether, the above results clearly indicated that death of dopaminergic cells, due to expression of m*GBA1* variant, was alleviated by the co-expression of a m*LRRK2* variant.

**Figure 6:**
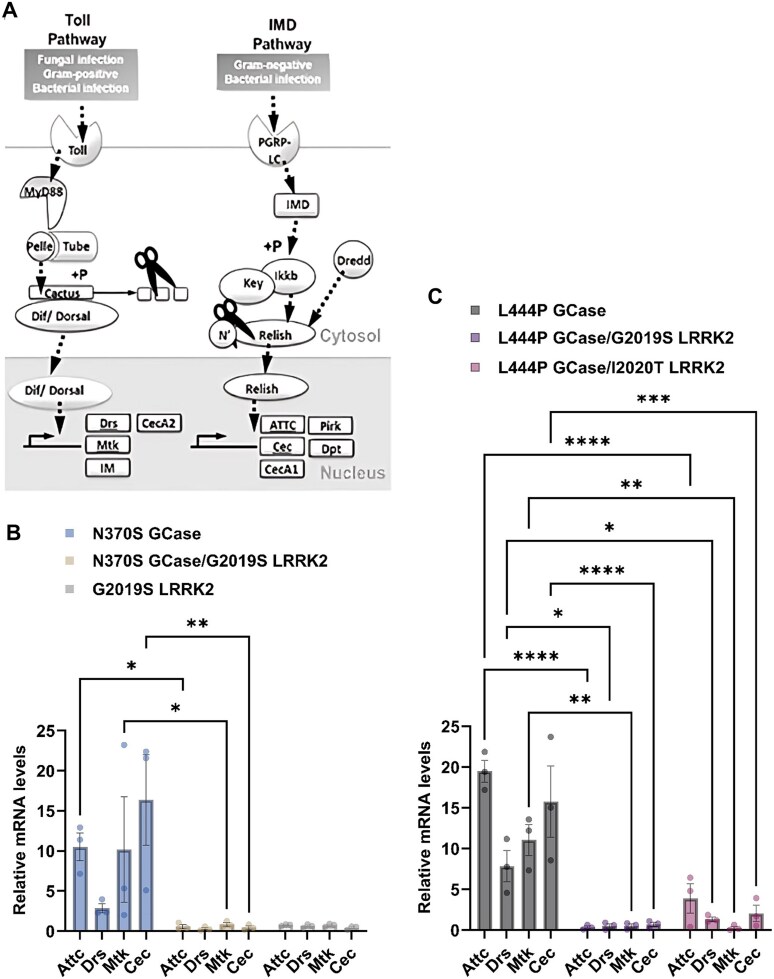
Neuroinflammation is decreased in flies co-expressing m*GBA1* and m*LRRK2.* (A) Schematic representation of the IMD and the Toll innate immunity pathways in *Drosophila* [71]*.* (B) mRNA levels of Attacin-C (ATTC), Drosomycin (Drs), Metchnikowin (Mtk) and Cecropin (Cec) in heads of flies at day 22 post-eclosion, expressing N370S GCase (blue bars), N370S GCase and G2019S LRRK2 (brown bars), G2019S LRRK2 (white bars), were analyzed by qRT-PCR. Expression was under a Ddc-GAL4 driver. The results are the mean ± SEM of three independent experiments. (C) Same as B, for L444P GCase (grey bars), L444P GCase and G2019S LRRK2 (purple bars) and L444P GCase and I2020T LRRK2 (pink bars) flies. Two-way ANOVA was used to calculate the significance of the results: * < 0.05, ** < 0.01, *** < 0.001, **** < 0.0001.

Neuroinflammatory mechanisms have been identified in recent years as both a driving force and a result of neurodegeneration seen in PD [80]. In *Drosophila*, there are two major pathways associated with immune response activation, the Toll and the Imd pathways, which are homologous to mammalian Toll-like receptor (TLR) and tumor necrosis factor receptor (TNFR) pathways, respectively [81]. Activation of these receptors leads to signaling pathways that initiate transcription of antimicrobial peptide (AMP) genes (Fig. 6A). To test neuroinflammation, mRNA levels of four different AMP genes were examined: Attacin-C (ATTC) and Cecropin (Cec), linked to the IMD pathway, and Drosomycin (Drs) and Metchnikowin (Mtk), associated with the Toll pathway. A significant increase in expression level of all tested genes was noticed in 22-days old flies expressing human m*GBA1* variant (Fig. 6B and C). However, in flies expressing both a m*GBA1* variant and the G2019S m*LRRK2* variant, there was a significant decrease in the expression level of the tested AMP genes, strongly suggesting a modifying effect of the m*LRRK2* gene on the mutant *GBA1* gene. Flies expressing the G2019S *LRRK2* variant did not exhibit elevation in expression of the four AMP genes tested in 22-days old flies (Fig. 6B and C), as has been previously shown [82] and likely reflecting the milder nature of *LRRK2*-associated PD.

**Figure 7:**
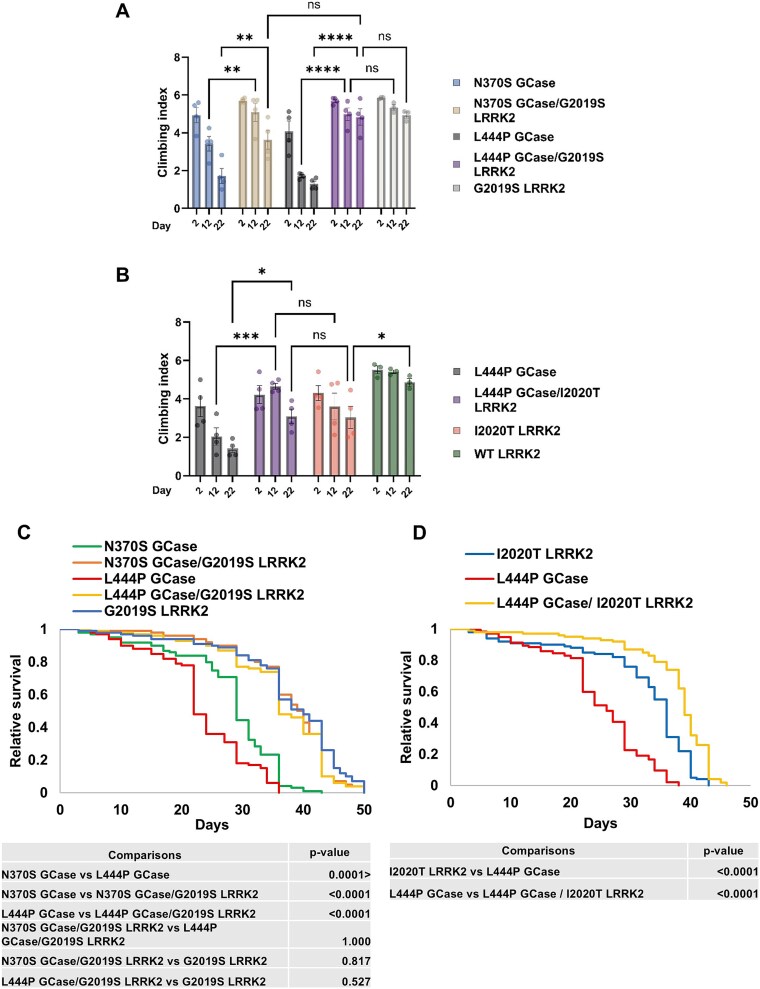
Locomotion and survival are improved in flies co-expressing m*GBA1* and m*LRRK2.* (A) Thirty flies expressing either single mutant *GBA1* (N370S (blue bars) or L444P (grey bars)), or single G2019S *LRRK2* (white bars) or both mutant *GBA1* and mutant G2019S *LRRK2* (brown and purple bars for N370S and L444P, respectively) were analyzed for locomotion behavior at days 2, 12 and 22 post-eclosion. Expression was under a Ddc-GAL4 driver. The value obtained for 2-days old flies for each genotype was considered 1. The results are the mean ± SEM of five independent experiments. Two-way ANOVA was used to calculate the significance of the results (N370S: Day 12 p = 0.0099, day 22 p = 0.0022; L444P: Day 12 p < 0.0001, day 22 p < 0.0001). (B) Same as (A), using flies expressing L444P *GBA1* (grey bars), I2020T *LRRK2* (pink bars), WT *LRRK2* (green bars) or both L444P *GBA1* and G2019S *LRRK2* (purple bars)*.* The results are the mean ± SEM of five independent experiments. Two-way ANOVA was used to calculate the significance of the results (day 12 p = 0.0006, day 22 p = 0.0385). (C) Kaplan Meier curve showing the overall survival rates of flies expressing either single mutant *GBA1* (N370S (green line) or L444P (red line)), single mutant G2019S LRRK2 (blue line) or both mutant *GBA1* and mutant G2019S *LRRK2* (orange and yellow lines for N370S and L444P, respectively). (D) Same as (C), using flies expressing L444P *GBA1* (red line), I2020T *LRRK2* (blue line) or both (yellow line).

Patients with PD commonly exhibit movement disturbances like bradykinesia and dystonia [5]. To assess movement in flies, we followed their age-dependent negative geotaxis, which reflects climbing ability [83]. Climbing was monitored at 2-, 12- and 22-days post-eclosion. An age-dependent decrease in the climbing ability of mGCase expressing flies was noted, and was significantly improved in the presence of mLRRK2, across all tested mutation combinations (Fig. 7A and B). No significant decline in climbing ability was noted in WT LRRK2 expressing flies at the tested ages.

The effect of mLRRK2 on survival of mGCase expressing flies was tested at 29°C, under the assumption that the added stress of the higher temperature [84] will further increase m*LRRK2* effect. Indeed, survival of the mGCase expressing flies was significantly shorter than that of the mLRRK2 expressing flies (Fig. 7C and D), recapitulating the more severe clinical course typically observed in *GBA1*-associated PD compared to *LRRK2*-linked PD in humans [85]. Notably, flies expressing both m*GBA1* and m*LRRK2* exhibited a significantly longer lifespan compared to flies expressing only m*GBA1*, across all mutations tested (Fig. 7C and D).

In conclusion, the results strongly suggested that the presence of a mutant *LRRK2* variant mitigates the detrimental effect of m*GBA1* variant in the flies.

### The presence of active LRRK2 is essential for its positive effect on mGCase

**Figure 8:**
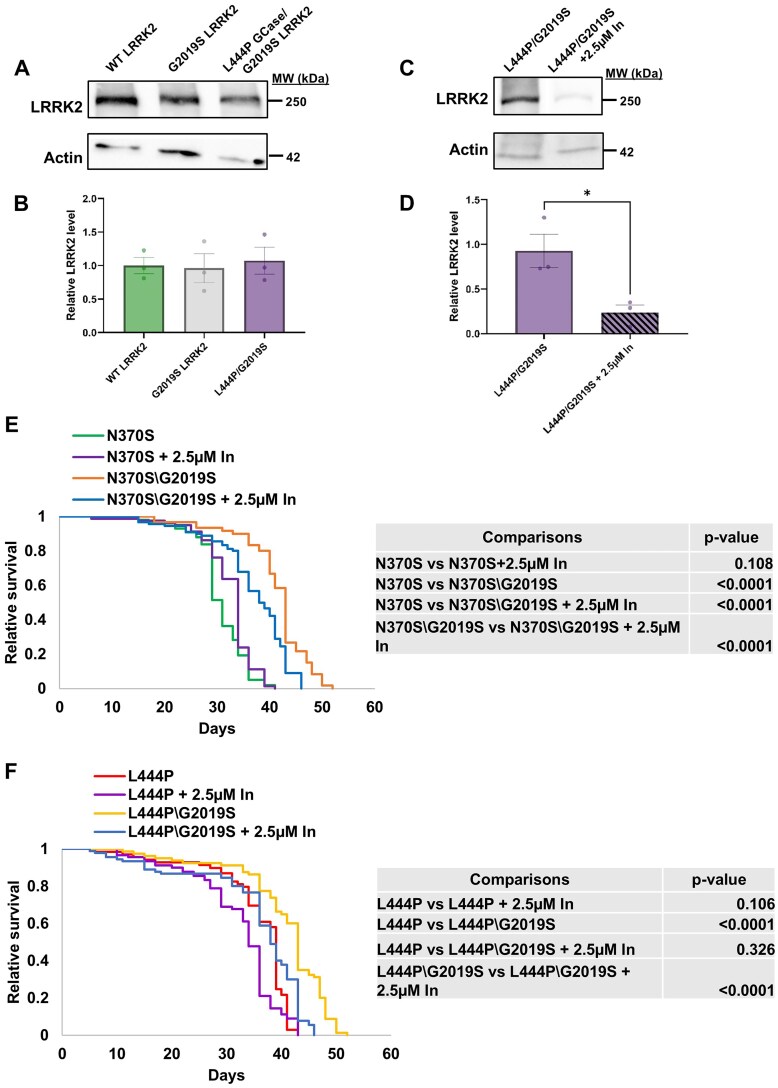
The presence of active LRRK2 is essential for its positive effect on mGCase. (A) Protein lysates were prepared from 10 flies expressing different human LRRK2 variants (WT LRRK2 (green bar), G2019S LRRK2 (white bar), and L444P GCase/G2019S LRRK2 (purple bar)) and subjected to 6% SDS-PAGE and western blotting. The corresponding blots were interacted with anti-LRRK2 antibodies. As a loading control, the blots were interacted with anti-actin antibody. Expression was under a Da-GAL4 driver. (B) Intensities of the corresponding bands as presented in (A) were quantified by densitometry and the LRRK2 amount was divided by that of actin at the same lane. The value obtained for WT LRRK2 flies was considered 1. The results are the mean ± SEM of three independent experiments. One-way ANOVA analysis was used to calculate the significance of the results. (C) Protein lysates were prepared from 10 flies expressing L444P GCase/G2019S LRRK2, treated (hatched bar) or untreated (purple bar) with 2.5 μM LRRK2-IN-1, and subjected to 6% SDS-PAGE and western blotting. The corresponding blots were interacted with anti-LRRK2 antibodies. As a loading control, the blots were interacted with anti-actin antibody. Expression was under a Da-GAL4 driver. (D) Intensities of the corresponding bands as presented in (C) were quantified by densitometry and the LRRK2 amount was divided by that of actin at the same lane. The value obtained for untreated flies was considered 1. The results are the mean ± SEM of three independent experiments. Unpaired student’s t-test analysis was used to calculate the significance of the results (p = 0.0283). (E) Kaplan Meier curve showing the overall survival rates of flies expressing either N370S *GBA1* grown with (purple line) or without (green line) 2.5 μM LRRK2-IN-1, as well as N370S *GBA1* with G2019S *LRRK2* grown with (blue line) or without (orange line) 2.5 μM LRRK2-IN. (F) Kaplan Meier curve showing the overall survival rates of flies expressing either L444P *GBA1* grown with (purple line) or without (red line) 2.5 μM LRRK2-IN-1, as well as L444P *GBA1* with G2019S *LRRK2* grown with (blue line) or without (yellow line) 2.5 μM LRRK2-IN.

The presented results indicated that expression of mLRRK2 modifies the severity of m*GBA1*-associated parkinsonian signs in the fly. In line with the clinical observations [62–64] and the presented results, we assumed that inhibition of mutant LRRK2 in double mutant flies will reverse the mLRRK2 positive effect. To test this assumption, LRRK2-IN-1, a potent and selective LRRK2 inhibitor, which causes dephosphorylation, ubiquitination and degradation of both mutant and WT LRRK2 [86] was employed. Treatment of *Drosophila* with this inhibitor was proven to be effective in lowering LRRK2 levels [56]. Protein levels of normal and mutant human *LRRK2* transgenes in the flies were tested (Fig. 8A and B), along with the reduction in LRRK2 levels upon treatment with the inhibitor (Fig. 8C and D). We observed a comparable expression level of WT or G2019S (with or without L444P GCase) and a significant decrease in LRRK2 level following treatment with the inhibitor.

Treating double mutant flies expressing the G2019S *LRRK2* variant and a *GBA1* variant with the inhibitor, instigated a significant drop in longevity, both when in combination with the mild N370S (Fig. 8E) or the severe L444P (Fig. 8F) *GBA1* variants. Still, in the case of the N370S variant, the LRRK2 inhibitor did not shorten the survival of the double mutant flies to the level of the corresponding single mGCase flies. Treatment of G2019S LRRK2 expressing flies with the inhibitor led to improved survival rates (Fig. S4), as was previously described by Liu et al, who used different LRRK2 inhibitors [87].

To summarize, the presented results provided further evidence that expression of human m*LRRK2* variant in the dopaminergic cells of the fly contributes to improvement of m*GBA1*-associated parkinsonian-like signs.

### Mutant GCase protein level is decreased in flies expressing mLRRK2

**Figure 9:**
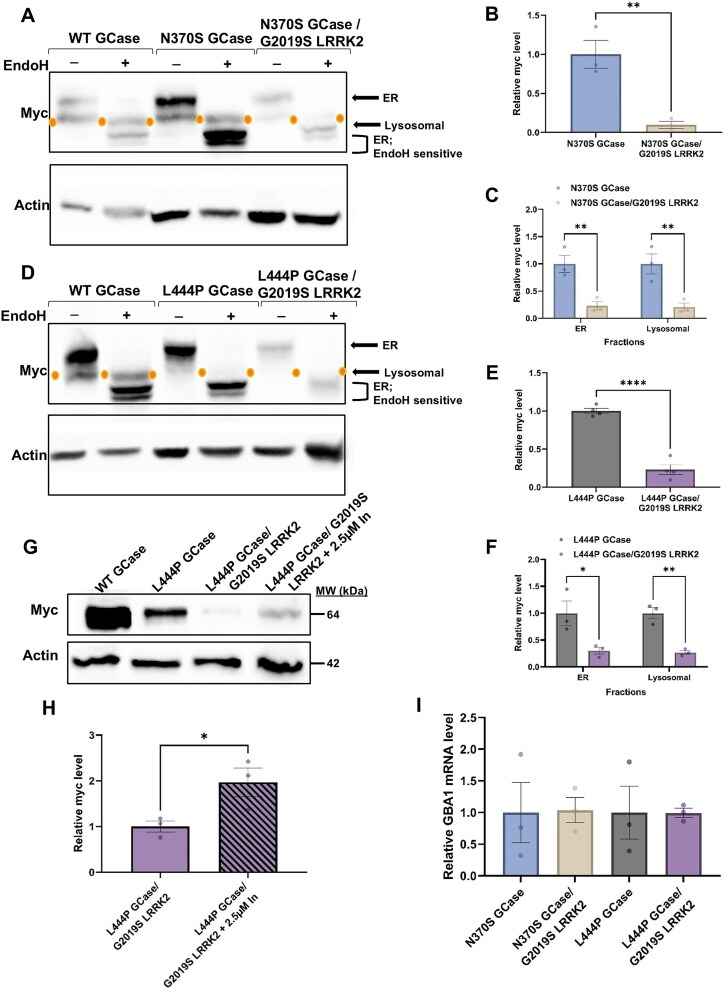
The steady state level of mGCase reduces in the presence of mLRRK2. (A) Protein lysates were prepared from 10 flies expressing the human WT GCase, N370S GCase (blue bar), and both N370S GCase and G2019S LRRK2 (brown bar) at 2-days post-eclosion. Samples were subjected to overnight endoH digestion, western blotting and the corresponding blots were interacted with anti-myc antibody. As a loading control, the blots were interacted with anti-actin antibody. (B) Intensities of the corresponding bands in the untreated lanes as shown in (A) were quantified by densitometry and GCase amount was divided by that of actin at the same lane. The value obtained for untreated N370S GCase was considered 1. The results are the mean ± SEM of three independent experiments (paired student’s t-test: *P* = 0.0081). (C) Same as (B), summarizing myc level in different cellular fractions, as shown in (A). The results are the mean ± SEM of three independent experiments. Two-way ANOVA analysis was used to calculate the significance of the results (ER *P* = 0.0067, lysosomal *P* = 0.0057). (D) Same as (A), using the L444P GCase variant, with (purple bar) and without (grey bar) mLRRK2. (E) Same as (B), quantifying the blot shown in (D). The value obtained for untreated L444P GCase was considered 1. The results are the mean ± SEM of four independent experiments (paired student’s t-test: *P* < 0.0001). (F) Same as (E), summarizing myc level in different cellular fractions, as shown in (D). The results are the mean ± SEM of three independent experiments. Two-way ANOVA analysis was used to calculate the significance of the results (ER *P* = 0.0107, lysosomal *P* = 0.0082) (G) protein lysates were prepared from 22-days post-eclosion flies expressing either WT GCase, L444P GCase or from double mutant flies L444P GCase/G2019S LRRK2 flies with or without 2.5 μM of LRRK2-IN treatment. Lysates were subjected to western blotting and the corresponding blots were interacted with anti-myc antibody. As a loading control, the blots were interacted with anti-actin antibody. Expression was under a Da-GAL4 driver. (H) Intensities of double mutant flies with (hatched bar) and without (purple bar) treatment corresponding bands as shown in (E) were quantified by densitometry and GCase amount was divided by that of actin at the same lane. The value obtained for double mutant untreated flies was considered 1. The results are the mean ± SEM of three independent experiments (paired student’s t-test: *P* = 0.0454). I mRNA levels of *GBA1* in flies expressing either mutant *GBA1* (blue and grey bars) or both mutant *GBA1* and *LRRK2* (brown and purple bars) were analyzed by qRT-PCR*.* The results are the mean ± SEM of three independent experiments. One-way ANOVA was used to calculate the significance of the result. Expression was under a Ddc-GAL4 driver.

Thus far, we have shown that flies expressing human mGCase and human mLRRK2 present decreased UPR parameters and a milder parkinsonian-like phenotype as evidenced by brain TH levels, neuroinflammation, movement and survival, in comparison to flies expressing human mGCase alone. Previous studies have shown that removal of mutant human GCase from the ER by the GCase chaperone ambroxol [88] alleviates the *GBA1*-induced parkinsonian-like phenotype in flies [43, 46]. This led us to investigate whether the mitigating effect of mLRRK2 on mGCase might be attributed to reduced ER stress, potentially via enhanced removal of mutant GCase from the ER. In order to test a possible change in localization of mGCase in the presence of mLRRK2 we employed the endonuclease-H (endoH) sensitivity assay [38, 44]. EndoH is a specific endo-glycosidase that selectively cleaves high mannose N-glycan complexes with more than four residues, while sparing mature counterparts [89]. High mannose structures, containing 8–9 mannose residues in the N-glycan trees, are predominantly found on glycoproteins within the endoplasmic reticulum (ER), making this fraction of glycoproteins sensitive to endoH. However, as glycoproteins progress to the cis-Golgi network, five mannose residues are enzymatically removed from their N-glycans, resulting in only four to three mannoses remaining on the N-glycan tree [90]. Thus, the fraction of glycoproteins localized in lysosomes is endoH resistant. In previous studies we demonstrated that the severity of GCase mutations is reflected by their endoH sensitive, ER-retained fraction which was measured in GD patients’ skin fibroblasts [38] and in peripheral blood mononuclear cells [91]. The more severe a mutation was—the larger the endoH sensitive, ER-retained fraction it presented. These results were recapitulated in flies expressing human N370S or L444P *GBA1* variants [44]. Given these findings, the ER fraction of mGCase was tested in protein extracts prepared from 2-days old flies expressing different mGCase variants alone or with mLRRK2, using the endoH sensitivity assay. The results indicated that flies co-expressing mGCase and G2019S LRRK2 exhibited approximately 20% of the GCase levels observed in flies expressing mGCase alone (Fig. 9A–F; Fig. S5 shows the results obtained for 22-days old flies). The relative levels of ER-retained and mature lysosomal mGCase remained largely unchanged in the double mutant flies compared to those observed in flies expressing only mGCase (Fig. 9C and F). This indicated a general decrease in the steady state level of mGCase in flies co-expressing both mGCase and mLRRK2. To confirm that the presence of mLRRK2 is responsible for the significant decrease in the steady state level of mGCase, flies expressing mGCase and mLRRK2 were treated with the LRRK2-IN-1 inhibitor. As expected, a two-fold increase in the amount of GCase in the LRRK2-IN-1 treated double mutant flies was observed compared to untreated age-matched flies (Fig. 9G and H), indicating the direct involvement of mLRRK2 in the decreased level of mGCase in the double mutant flies.

To confirm that the observed phenotype in flies carrying both mGCase and mLRRK2, compared to those carrying only mGCase, is not due to reduced expression of the m*GBA1* transgene in the double mutant fly lines, we measured *GBA1* mRNA levels in 2-days old flies. The results showed no significant differences in m*GBA1* expression in the absence or presence of m*LRRK2* (Fig. 8I). This strongly suggested that the expression level of m*GBA1* in the presence of m*LRRK2* does not account for the modifying effect of mLRRK2 on mGCase.

Altogether, the results strongly indicated that the presence of mLRRK2 is responsible for the decreased steady state level of mGCase in the double mutant flies, leading to ameliorated parkinsonian-like signs.

### UPR in PD patient derived fibroblasts

Previous results from our lab showed elevation in transcription of UPR related genes in cells that derived from carriers of different *GBA1* mutations [91]. Based on our findings in *Drosophila*, we extended our analysis to compare UPR parameters in cells from PD patients who carry mutations in either gene or in both *GBA1* and *LRRK2* genes. To this end, we tested mRNA levels of sXBP1, BiP, ATF4, ATF6 and CHOP, along with phosphorylation of eIF2α in fibroblasts that originated from one N370S *GBA1*/G2019S *LRRK2* PD patient, one N370S *GBA1* PD patient and three G2019S *LRRK2* PD patients. Our results showed significantly decreased expression of UPR related genes in skin fibroblasts of the double mutant patient, compared to the N370S *GBA1* patient (Fig. S6 and S7). There was no significant increase in 4 out of 5 tested UPR parameters in cells that derived from PD patients carrying a G2019S *LRRK2* variant.

The results presented in this section strongly indicated ameliorated UPR in a patient with N370S *GBA1* variant and G2019S *LRRK2* variant in comparison to a patient with only a N370S *GBA1* variant.

In conclusion, our findings clearly indicate that mLRRK2 modulates mGCase-associated PD by reducing the cellular steady-state levels of mutant GCase, suppressing the UPR in both flies and human skin fibroblasts, and alleviating parkinsonian-like symptoms in flies. These effects were evidenced by decreased neuroinflammation, an increased number of dopaminergic neurons, improved motor function, and enhanced survival.

## Discussion

The goal of this study was to investigate the potential genetic interaction between human m*GBA1* and human m*LRRK2* in *Drosophila* flies, based on the assumption that m*LRRK2* modifies the effect of *GBA1*-associated PD. Given the dominant nature of m*LRRK2* and m*GBA1*-associated PD, we employed a *Drosophila* model system to co-express various human *GBA1* and *LRRK2* transgenes, acknowledging the overexpression inherent to this approach. This system has been widely used to study neurodegenerative diseases with gain-of-function expression like PD [35, 46, 56], Alzheimer disease [92, 93] and amyotrophic lateral sclerosis (ALS) [94, 95].

Given our previous findings that expression of mutant GCase in dopaminergic neurons of flies activates the UPR and leads to the development of parkinsonian symptoms [43, 44], we sought to test whether co-expression of m*LRRK2* variants could alleviate these symptoms, as suggested by clinical studies [62–64]. It is important to note that our system was not designed, nor was it appropriate, to assess any contribution of glucosylceramide or glucosylsphingosine to PD development.

The results strongly indicated that in double mutant flies expressing m*GBA1* and m*LRRK2* genes, compared to m*GBA1* expressing flies, there was a significant decrease in the steady state level of mutant GCase with the same effect on ER and lysosomal fractions. This reduction likely reflects an enhanced exit of mutant GCase from the ER to the lysosomes and enhanced degradation of the mutant enzyme in the lysosomes, concluding in lessened UPR and alleviation of parkinsonian signs as manifested by diminished neuroinflammation and increase in TH levels (less death of dopaminergic cells), motoric skills and survival. The modifying effect of mLRRK2 is reminiscent of the effect pharmacological or chemical chaperones (ambroxol or arimoclomol, respectively) exert in mGCase expressing flies, in respect to decreased UPR and improvement in parkinsonian symptoms [96, 97]. In the present project we did not test which specific function of mLRRK2, its kinase activity, GTPase activity, or another function—is responsible for this effect. We assume that mLRRK2 plays a critical role in enhancing intracellular trafficking from the ER to lysosomes [49, 57, 59].

A previous study by Kedariti et al [98] investigated the potential interaction between mGCase and mLRRK2 using various models, including fibroblasts from PD patients, patient iPSC-derived neurons, and mouse brain tissue. The study found that GCase protein level was reduced in mouse *LRRK2* G2019S knock-in brain tissues and in G2019S iPSC-derived neurons but increased in fibroblasts and peripheral blood mononuclear cells (PBMCs) from *LRRK2* G2019S carriers. They also showed that both pharmacological and genetic inhibition of LRRK2 kinase activity led to decreased GCase activity. Overall, the authors concluded that while the interaction between these two genes is generally positive, the outcomes vary depending on the model, cell type, or specific mutation examined.

Our results raise an imperative question: how does presence of mLRRK2 lead to change in the steady state level of intracellular GCase? Theoretically, mLRRK2 may a) have an impeding effect on the rate of mGCase protein synthesis, or b) it may affect the degradation rate of mGCase, or c) it may cause the removal of mGCase from the cell.

Inhibition of protein translation by LRRK2 was documented in G2019S LRRK2 PD-patient derived fibroblasts, showing a 40% decrease in global protein synthesis [99]. However, work done by Martin et al demonstrated that expression of transgenic human G2019S *LRRK2* causes an increase in bulk translation in *Drosophila* [100]. Since the two publications describe different models, it remains unclear whether arrest in protein translation can serve as a possible explanation for the decrease in mGCase steady state level in double mutant flies and should be further investigated.

Another mechanism, by which mLRRK2 may cause the decrease in GCase steady state level, is protein degradation. The two main cellular mechanisms responsible for protein turnover are the ubiquitin-proteasome system (UPS) and the lysosomal proteolysis pathway [101]. Our lab has shown in the past that mGCase is degraded by the proteosome machinery, in an attempt to remove the mutant protein retained in the ER and restore cellular homeostasis [38]. mLRRK2 may potentially increase the rate of mGCase ERAD, allowing for clearance of retained misfolded protein from the ER and its degradation in the proteasome. However, using different tissue culture lines (MEFs, HeLa), it was shown that LRRK2 overexpression may actually inhibit clearance of proteasomal substrates [102].

Lysosomal degradation of mutant proteins has been documented in the literature [103]. Import of mutant misfolded proteins to the lysosomes for degradation may occur through the autophagic pathway [104] or by direct vesicular trafficking from other intracellular organelles [105]. Lysosomal degradation of mutant GCase was suggested by Sulzer et al., who documented that mutant GCase was localized to lysosomal surfaces in samples from postmortem human *GBA1*-PD brains. The authors argued that transport of mutant GCase to the lysosomes occurs through chaperone-mediated autophagy (CMA) following retrotranslocation form the ER, as part of its ERAD [106]. Several publications indicated the participation of LRRK2 in autophagy related processes. One study documented that G2019S LRRK2 kinase activity leads to an increase in the number of autophagosomes in human cell lines, activated through AMPK pathway [107]. Using an *in vivo* mouse model, another study showed that loss of LRRK2 led to impaired autophagy-lysosomal pathway and dramatically increased apoptotic cell death, inflammatory responses, and oxidative damage in mice [108], arguing that LRRK2 mutations may also cause impairment of protein degradation pathways. Increased rate of lysosomal degradation may also be a result of elevation in rate of vesicular intracellular trafficking from the ER or from the Golgi apparatus to the lysosomes. Such enhanced trafficking of mGCase from the ER to the lysosomes would result in increased rate of its lysosomal degradation. A study conducted by Alcalay et al demonstrated that in dried blood spots from mLRRK2 PD patients there was elevated activity of various lysosomal enzymes [65], suggesting that presence of mLRRK2 promotes enhanced trafficking to the lysosomes.

Another potential pathway for GCase degradation, recently discussed in the literature, involves mitochondria. Baden et al. [109] showed that GCase can be imported from the cytosol into the mitochondria via recognition of internal mitochondrial targeting sequence-like signals. Within the mitochondria, GCase played a crucial role in maintaining the integrity and function of mitochondrial complex I. However, disease-associated mutations impaired complex I stability and function. Notably, mitochondrial GCase undergoes degradation within the mitochondria. In the present study, we focused on two cellular forms of GCase—the ER and lysosomal forms—but it is possible that the ER-associated form also includes a mitochondrial fraction.

In recent years, Rab GTPases have emerged as *bona-fide* LRRK2 phosphorylation substrates, and their involvement in intracellular trafficking has implicated them in the development of PD [109, 110]. Rab proteins regulate the transport and arrangement of vesicles within the different intracellular vesicular pathways, among them the lysosomal and the endocytic pathways [111]. For example, Rab8 and Rab10 undergo LRRK2 phosphorylation and pathogenic LRRK2 mutations enhance their phosphorylation [110, 112]. Such hyperphosphorylation stabilizes Rabs in their active GTP-bound state [113] and affects their cellular localization [114], which in turn can explain the increased rate of intracellular vesicular trafficking.

Another mechanism that may decrease cellular steady state level of mutant GCase is exosomal trafficking. Exosomes are small membrane vesicles, that are secreted extracellularly by many cell types [115]. They originate as internal vesicles of the endosomal compartment and are released upon exocytic fusion of these organelles with the plasma membrane. Intracellularly, they are formed by inward budding of the endosomal membrane in a process that sequesters particular proteins and lipids [116].

It is of note that a recent publication [47] has shown the existence of *GBA1* transcripts, mainly in the brain, which encode non-lysosomal *GBA1* proteins with no GCase activity. As mentioned by the authors, these novel *GBA1* isoforms, particularly those lacking GCase activity, may contribute to phenotypic diversity in GD and PD. Further experimentation would be necessary to validate this hypothesis. However, in the present study we used one human *GBA1* encoding sequence coupled to the yeast UAS and therefore we did not expect more than one GBA1 protein product, namely GCase.

LRRK2 kinase inhibition is currently being explored in clinical studies as a possible therapeutic prospect for non-idiopathic PD patients [117]. Our results strongly suggest that treatment with a LRRK2 inhibitor may not befit PD patients carrying mutations in both the *GBA1* and the *LRRK2* genes and might even worsen the severity of parkinsonian symptoms, as was previously addressed [63].

Another emerging therapeutic strategy for *GBA1*-associated PD is treatment with pharmacological chaperones, small molecules that bind to mutant proteins in the ER and stabilize their native folding, thus enabling them to leave the ER [118, 119]. However, given the observed decrease in GCase levels in double mutant flies, this treatment strategy may also prove to be ineffective for carriers of mutations in both the *GBA1* and the *LRRK2* genes.

In conclusion, our findings strongly suggest that the presence of mLRRK2 in flies carrying mGCase leads to decreased steady state level of mGCase, thus causing reduced UPR and alleviated parkinsonian symptoms. We could recapitulate decreased UPR parameters in skin fibroblasts from a PD patient, heterozygotes for N370S *GBA1* and G2019S *LRRK2*. The precise mechanism by which mutant LRRK2 causes the decreased steady state levels of GCase warrants further investigation.

## Materials and methods

### Flies

All experiments were performed in isogenized w^1118^ background obtained from the Bloomington Drosophila Stock Center, Indiana University, Bloomington. Transgenic flies, harboring pUAS-mycHis-N370SGCase and pUAS-mycHis-L444PGCase on the second chromosome, were described elsewhere [44, 120]. A strain harboring UAS-h*LRRK2*-I2020T was a kind gift of Prof. Hugo Bellen (Department of Molecular and Human Genetics, Baylor College of Medicine). Fly lines harboring the UAS-Flag-h*LRRK2*-G2019S was a kind gift of Prof. Christopher Elliott (Department of Biology, University of York, UK). *Gba1b*^m/m^ flies, described elsewhere [71], are homozygous for a Minos element insertion in their *Gba1b* gene, causing a 133 C-terminal amino acids deletion. Da-GAL4 (No. 55849) and Ddc-GAL4 driver lines (No. 7009) were from Bloomington Stock Center and were used to drive expression ubiquitously [121, 122] or in dopaminergic neurons [123], respectively. Strains were maintained on standard cornmeal-molasses medium at 25°C and experiments were conducted at either 25°C or 29°C. Additionally, to avoid any sex bias, all experiments were conducted with an equal number of males and females.

### Climbing assay of flies

Climbing behavior of adult flies was measured using a countercurrent apparatus. Groups of approximately 30 flies (both males and females) were given 10 min to adapt in the starting tube, and then 20 seconds to move upwards against gravity to the upper frame’s tube. The top frame of tubes was then shifted to the right so that the start tube came in contact with the second bottom tube and flies, which successfully climbed up, were tapped down again, falling into tube 2. The upper frame was then returned to the left and the flies were once again allowed to climb into the upper tube. After five repetitions, the number of flies in each tube was counted. For each time point, three cohorts from each genotype were scored.

### Survival assay

For each fly strain, 10 vials, each containing 5 males and 5 females, were maintained on food from day one post-eclosion at 29°. Fresh food was supplied every other day and deaths were recorded. Each panel in the results section represents an independent set of lines, carried out in parallel, and statistical comparisons are valid only within a set. Kaplan Meier analysis was performed using ExcelStat.

### Cell lines

Human primary skin fibroblasts originated from PD patients, who signed an informed consent (Sheba Medical Center). Identifiable clinical and personal data from the patients were not available for this study. Work with the cell lines was in accordance with Tel Aviv University institutional guidelines. Cells were grown in DMEM supplemented with 20% FBS (Biological Industries, Beit Haemek, Israel), at 37°C in the presence of 5% CO2.

### Enzymatic activity

Adult flies were lysed in McIlvaine’s buffer (0.1 M citric acid, pH 4.2, and 0.2 M Na2HPO4) and protein concentration was determined. Tissue homogenates containing 100 μg of protein were incubated for 1 h at 37°C with 8 μM of N-[6-[(7-Nitro-2-1,3-benzoxadiazol-4-yl) amino]caproyl]-glucosylceramide (C6-NBD-GlcCer) (Avanti Polar Lipids, Alabaster, AL, USA). Reactions were terminated by addition of three volumes of chloroform:methanol (2:1). Lipids were extracted and the lower phase was separated by Thin Layer Chromatography (TLC) (see below). TLC plates were developed using Amersham Imager 600 (Amersham, Buckinghamshire, United Kingdom).

### Separation of sphingolipids on TLC

Flies were lysed in 300 μl of distilled water and 900 μl of chloroform:methanol (2:1,) were added. Following centrifugation, the lower phase was isolated and dried. Twenty microliters of chloroform:methanol (2:1) were added and the samples were separated by TLC (Silica gel 60A plates; Sigma-Aldrich, St. Louis, MO, USA) in chloroform: butanol: ethyl acetate: 0.25% KCl: methanol (25:25:25:9:16, by volume). The TLC plates were developed with primulin reagent (Sigma-Aldrich, St. Louis, MO, USA).

### RNA preparation

**Flies:** Adult flies (bodies or heads) were frozen in liquid nitrogen and homogenized in TRIzol® Reagent (Life Technologies, Carlsbad, CA, USA). RNA extraction was according to the manufacturer’s instructions. **Cells:** Cells were washed three times in 1 × PBS, scraped off the plate, pelleted by centrifugation in 1.5 mL tubes, and stored at −80°C until further use. RNA extraction from cell pellets was also achieved using TRIzol® Reagent according to the manufacturer’s instructions (Life Technologies, Carlsbad, CA, USA).

### RT- PCR

One microgram of RNA was reverse-transcribed with MMLV reverse transcriptase (Promega Corporation, Madison, CA, USA), using oligo dT primer (IDT, Jerusalem, Israel) in a total volume of 25 μl of MMLV RT buffer (Promega Corporation, Madison, CA, USA), at 42°C for 60 min. Reactions were stopped by incubation at 70°C for 15 min.

### Quantitative real time PCR (qRT-PCR)

One and a half microliters of cDNA were used for real time PCR. PCR was performed using ‘power SYBR green QPCR mix reagent’ kit (Applied Biosystems, Foster City, CA, USA), using CFX Connect Real-Time PCR Detection System (Bio-Rad Laboratories, Hercules, California, USA). The reaction mixture contained 5 μl of SYBR green mix, 300 nM of forward primer and 300 nM of reverse primer, in a final volume of 10 μl. Thermal cycling conditions were: 95°C (3 min), and 40 cycles of 95°C (30 s), 60°C (20 s) and 72°C (20 s). The expression level was determined by the 2^(-ΔΔCT)^ method and normalized to *Rp49* gene in flies or *GAPDH* gene in PD-patient derived fibroblasts. All experiments were performed in at least three independent biological replicates and each with three technical replicates. All primers used for the PCR reactions are detailed in Table 1.

**Table 1:** Primers used in the present study. The table contains the sequence of all the primers used in the present work.

**Name**	**Primers for qRT-PCR**
***Gba1b***	F: 5′-AAGAACTTCCGGTGGAGCTA-3′
	R: 5′-CAATTCATTGTATGCCCAGGGT-3′
***hGBA1***	F: 5'-CATCCGGGTACCCATGGCCAGCTGTTG-3'
	R: 5'-TCGATCCCAGGAGCCTAGCCGCACAC-3'
***Lrrk***	F: 5'-CGGCCTATTTAAACGCCACAGCAA-3'
	R: 5'-AACTGAAGTGTTGCGCGAAGAACC-3'
***hLRRK2***	F: 5'-CCTCCAAGGGTTCCTTGGATC-3'
	R: 5'-CAAAATGTCAAAGACCTGGGCAG-3'
***sXbp1***	F: 5′-CCGAACTGAAGCAGCAACAGC-3′
	R: 5′-GTATACCCTGCGGCAGATCC-3′
***Hsc-70-3***	F: 5′-GCTGGTGTTATTGCCGGTCTGC-3′
	R: 5′-GATGCCTCGGGATGGTTCCTTGC-3′
***Atf4***	F: 5′-AGACGCTGCTTCGCTTCCTTC-3′
	R: 5′-GCCCGTAAGTGCGAGTACGCT-3′
***Atf6***	F: 5′-CGTAATTCCACGGAAGCCCAAC-3′
	R: 5′-CGACGGTAGCTTGATTTCTAGAGCC-3′
***ATTC***	F: 5′-CTGCACTGGACTACTCCCACATCA-3′
	R: 5′-CGATCCTGCGACTGCCAAAGATTG-3′
***Cec***	F: 5′-CATTGGACAATCGGAAGCTGGGTG-3′
	R: 5′-TAATCATCGTGGTCAACCTCGGGC-3′
***Drs***	F: 5′-AGTACTTGTTCGCCCTCTTCGCTG-3′
	R: 5′-CCTTGTATCTTCCGGACAGGCAGT-3′
***Mtk***	F: 5′-CATCAATCAATTCCCGCCACCGAG-3′
	R: 5′-AAATGGGTCCCTGGTGACGATGAG-3′
***Rp49***	F: 5′-TAAGAAGCGCACAAAGCACT-3′
	R: 5′-GGGCATCAGATATTGTCCCT-3′
***GAPDH***	F: 5′-CTCCTCCTGTTCGACAGTCA-3′
	R: 5′-GTTGACTCCGACCTTCACCT-3′
***CHOP***	F:5′-AGCGACAGAGCCAAAATCAG-3′
	R: 5′-TCTGCTTTCAGGTGTGGTGA-3′
***BiP***	F: 5′-CATCAAGTTCTTGCCGTTCA-3′
	R: 5′-ATGTCTTTGTTTGCCCACCT-3′
***ATF4***	F: 5′-GTTCTCCAGCGACAAGGCTA-3′
	R: 5′-ATCCTGCTTGCTGTTGTTGG-3′
***sXBP1***	F: 5′-TCTGCTGAGTCCGCAGCAG-3′
	R: 5′-GAAAAGGGAGGCTGGTAAGGAAC-3′

### Protein extraction

**Flies:** For each preparation, 10–15 flies were homogenized in 100 μl RIPA lysis buffer (50 mM Tris/HCL, 150 mM NaCl, 1 mM EDTA, 1% TritonX-100, 1% sodium deoxycholate, and 0.1% SDS) containing protease inhibitors (10 μg/ml Leupeptin, 10 μg/ml Aprotinin and 0.1 mM PMSF (Sigma-Aldrich, St. Louis, MO, USA)). **Cells:** Cells were washed three times in 1× PBS, scraped off the plate, pelleted by centrifugation in 1.5 ml tubes, and stored at −80°C until further use. The cells were lysed in 100 μl of the lysis buffer described above.

### Endonuclease-H (endoH) treatment

Samples of cell lysates, containing 100 μg of total protein, were subjected to an overnight incubation with endoH (New England Biolabs, Beverly, MA, USA), according to the manufacturer’s instructions. They were then electrophoresed through 10% SDS-PAGE and the corresponding blot was interacted with the appropriate antibodies.

### SDS-PAGE and western blotting

Samples containing the same amount of protein were electrophoresed through 10% SDS–PAGE (unless detailed otherwise) and electroblotted onto a nitrocellulose membrane (Thermo Fisher Scientific, Waltham, Massachusetts, USA), which was interacted with the appropriate antibodies. The blots were developed and analyzed by ChemiDoc™ XRS (Bio-Rad Laboratories, USA).

### Immunofluorescence

Brain dissections, fixation, and immunostaining were performed as described [124]. Dissected brains were fixed in 4% paraformaldehyde (BN15710, Bar Naor, Israel) in PBS for 20 minutes at room temperature. Samples were reacted with anti-TH (1:80, AB152, Millipore, Billeria, MA, USA) primary antibodies and Cy3-conjugated goat anti-rabbit (1:200, Jackson Immuno Research Laboratories, West Grove, PA, USA) secondary antibodies. The preparations were mounted in mounting medium with DAPI (E19–18, Origene, Rockville, USA). Slides were visualized using Leica SP8 confocal microscope and collected Z-projections were analyzed using Imaris 10.1.1.

### Antibodies for western blotting

The following primary antibodies were used in this study: mouse monoclonal anti-myc antibody (1:1000 for WB, Cell Signaling Technology, Beverly, MA, USA; anti-LRRK2 antibodies (1:1000 for WB, Cell Signaling Technology, Beverly, MA, USA; mouse monoclonal anti-actin antibody (1:1000 for WB, Sigma-Aldrich, Rehovot, Israel); rabbit polyclonal anti-TH antibodies (1:2000 for WB, AB152, Millipore, MA, USA); rabbit polyclonal anti-P-eIF2A antibodies (1:1000 for WB, Sigma-Aldrich, Rehovot, Israel); rabbit polyclonal anti-eIF2a antibodies (1:1000 for WB, Sigma-Aldrich, Rehovot, Israel); Secondary antibodies used were: horseradish peroxidase-conjugated goat anti-mouse antibodies (1:5000 for WB, Jackson ImmunoResearch Laboratories, West Grove, PA, USA); horseradish peroxidase-conjugated goat anti-rabbit antibodies (1:5000 for WB, Jackson ImmunoResearch Laboratories, West Grove, PA, USA).

### Pharmacological treatments

Flies were treated with LRRK2-IN-1 inhibitor (2.5 μM, ab254524, Abcam, Cambridge, UK). Eighty μl of 2.5 μM LRRK2-IN-1 were pipetted on top of fresh food containing tubes, which were kept overnight at room temperature before use. The flies were moved to new tubes every other day.

### Statistical analysis

All statistical analyses were performed using GraphPad Prism 10.3.0, as described in the figure legends. All N numbers represent biological replicates. Data were pooled from 3 to 5 independent experiments. Students’ t-test, one-way ANOVA with Dunnett correction and two-way ANOVA with Holm Sidak correction were used as specified in figure legends. P values are represented as * = *P* < 0.05, ** = *P* < 0.01, *** = *P* < 0.001, **** = *P* < 0.0001. *P* < 0.05 was considered significant. Data are expressed as mean ± SEM or the median.

## Supplementary Material

supp_figures_revision_FINAL_ddaf062
