# Correction to: The modifying effect of mutant *LRRK2* on mutant *GBA1*-associated Parkinson disease

**DOI:** 10.1093/hmg/ddaf112

**Published:** 2025-06-24

This is a correction to: Vera Serebryany-Piavsky, Lian Egulsky, Julia Elia Manoim-Wolkovitz, Saar Anis, Sharon Hassin-Baer, Moshe Parnas, Mia Horowitz, The modifying effect of mutant LRRK2 on mutant GBA1-associated Parkinson disease, *Human Molecular Genetics*, 2025, ddaf062, https://doi.org/10.1093/hmg/ddaf062

In the originally published version of this manuscript, the authors' first and last names were erroneously transposed.

This has been corrected.

