# Supplementary material for: The modifying effect of mutant LRRK2 on mutant GBA1-associated Parkinson disease
Source: Hum Mol Genet. 2025 May 2;34(14):1184–203. doi: 10.1093/hmg/ddaf062 (PMC12228092; doi:10.1093/hmg/ddaf062)
Supplement: supp_figures_revision_FINAL_ddaf062 [file supp_figures_revision_final_ddaf062.pdf]

Supplementary Figure 1.

A

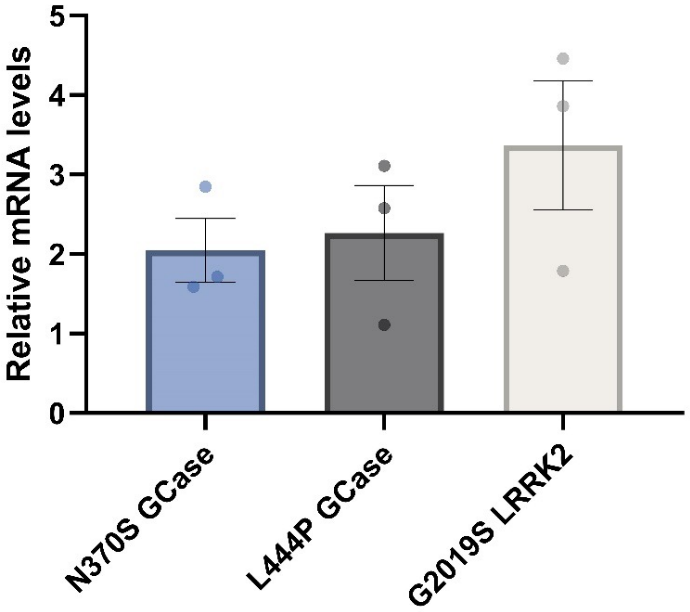

B

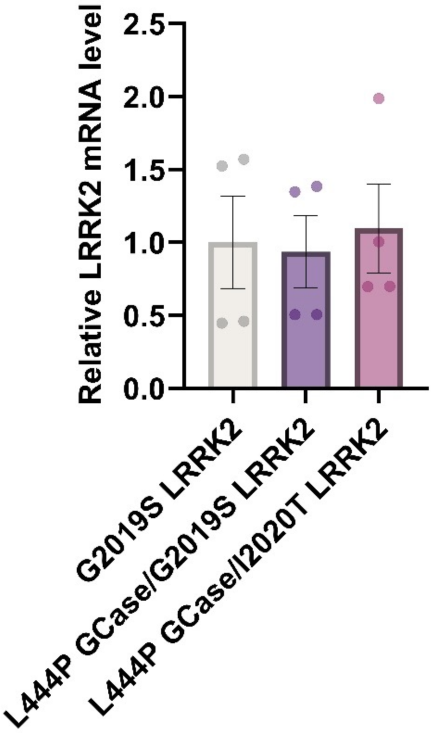

Figure S1. Expression levels of different transgenes. A mRNA levels of *GBA1* and *LRRK2* were evaluated using qRT-PCR analysis in flies expressing either m*GBA1* (blue and grey bars) or m*LRRK2* (white bars), with no significant differences found. The results are the mean ± SEM of three independent experiments. Expression was under a Ddc-GAL4 driver. **B** mRNA levels of *LRRK2* were evaluated using qRT-PCR analysis in flies expressing either single m*LRRK2* (white bars) or both m*GBA1* and m*LRRK2* (purple and pink bars), with no significant differences found. The results are the mean ± SEM of three independent experiments. Expression was under a Ddc-GAL4 driver.

Supplementary Figure 2.

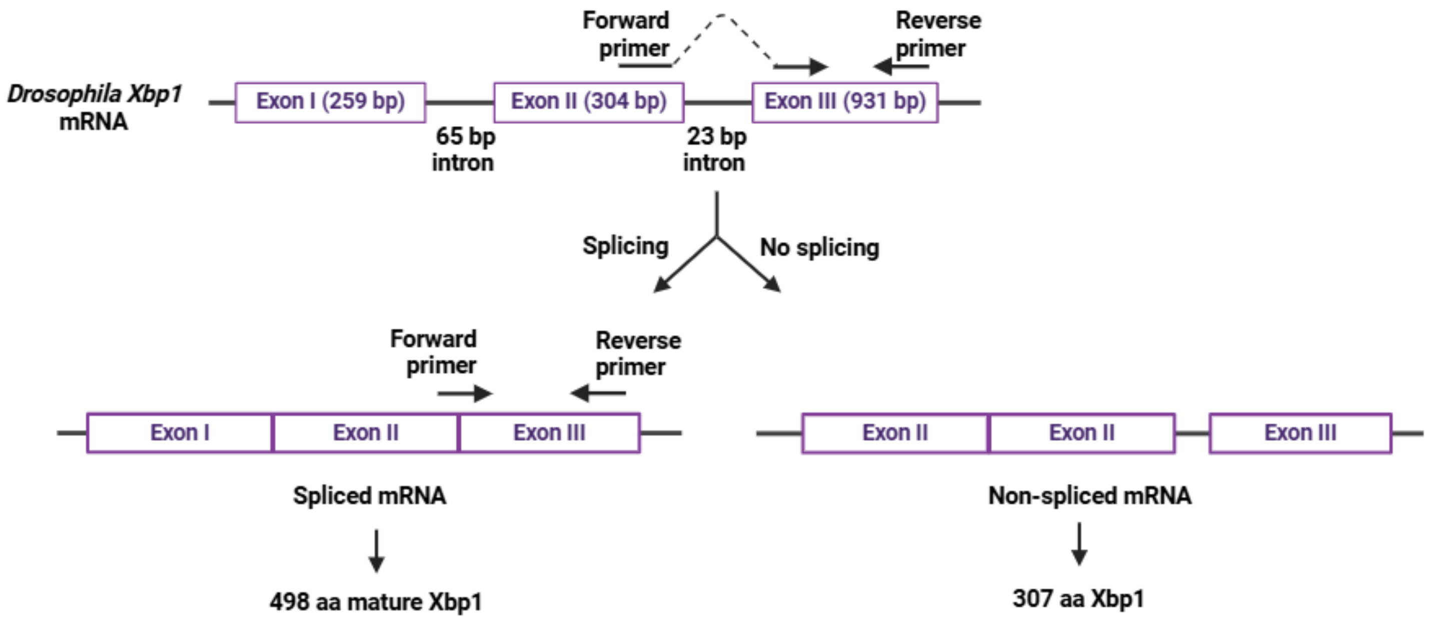

Figure S2. Detection of spliced Xbp1 mRNA by quantitative real time PCR. Schematic representation (not in scale) of the two predicted *Xbp1* mRNA isoforms. After splicing of the 23 bp 2nd intron, the spliced form encodes a 498 aa protein, while the non-spliced isoform encodes a 307 aa inactive protein. Also shown, the primers used for qRT-PCR of the spliced isoform. The forward primer can anneal only to the spliced form of *Xbp1* mRNA (created with BioRender) .

Supplementary Figure 3.

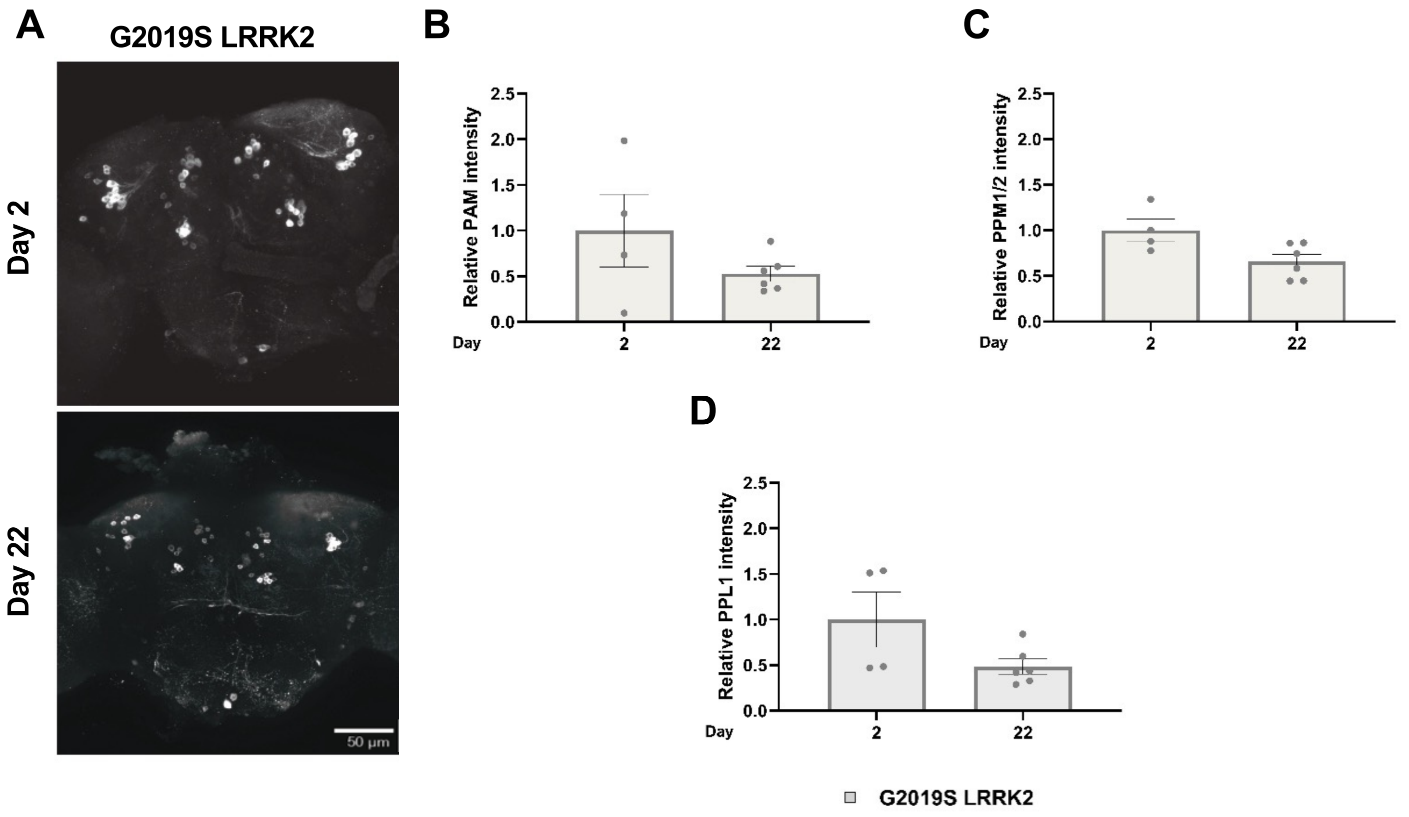

Figure S3. Dopaminergic neurons labeling in G2019S LRRK2 expressing flies. A Maximum intensity projection of 138,199 (top to bottom respectively, 0.5µm), confocal sections through the central brain of adult *Drosophila* carrying G2019S LRRK2 mutation following their staining with anti-TH antibody, at the ages of 2- and 22-days post-eclosion. **B-D** Quantification of the anti-TH signal intensity obtained from the brains described in (a) The results represent the mean ± SEM of 4-6 brains in each cluster, i.e. PAM (B), PPM1/2 (C) and PPL1 (D).

Supplementary Figure 4.

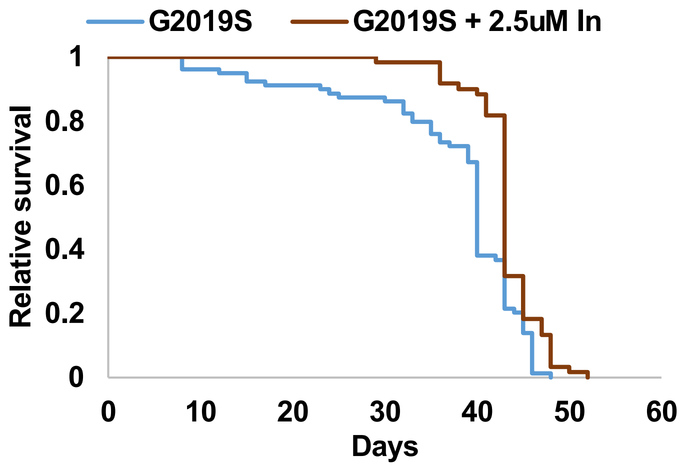

Comparisons	p-value
G2019S LRRK2 vs G2019S LRRK2 + 2.5µM In	0.001

Figure S4. LRRK2 inhibition. Kaplan Meier curve showing the overall survival rates of flies expressing the *LRRK2* mutation G2019S, with (brown line) and without (blue line) 2.5µM LRRK2-IN-1. Flies were grown 10 flies per vial at 29°C and were transferred to fresh food and counted every other day. Expression was under a Ddc-GAL4 driver.

Supplementary Figure 5.

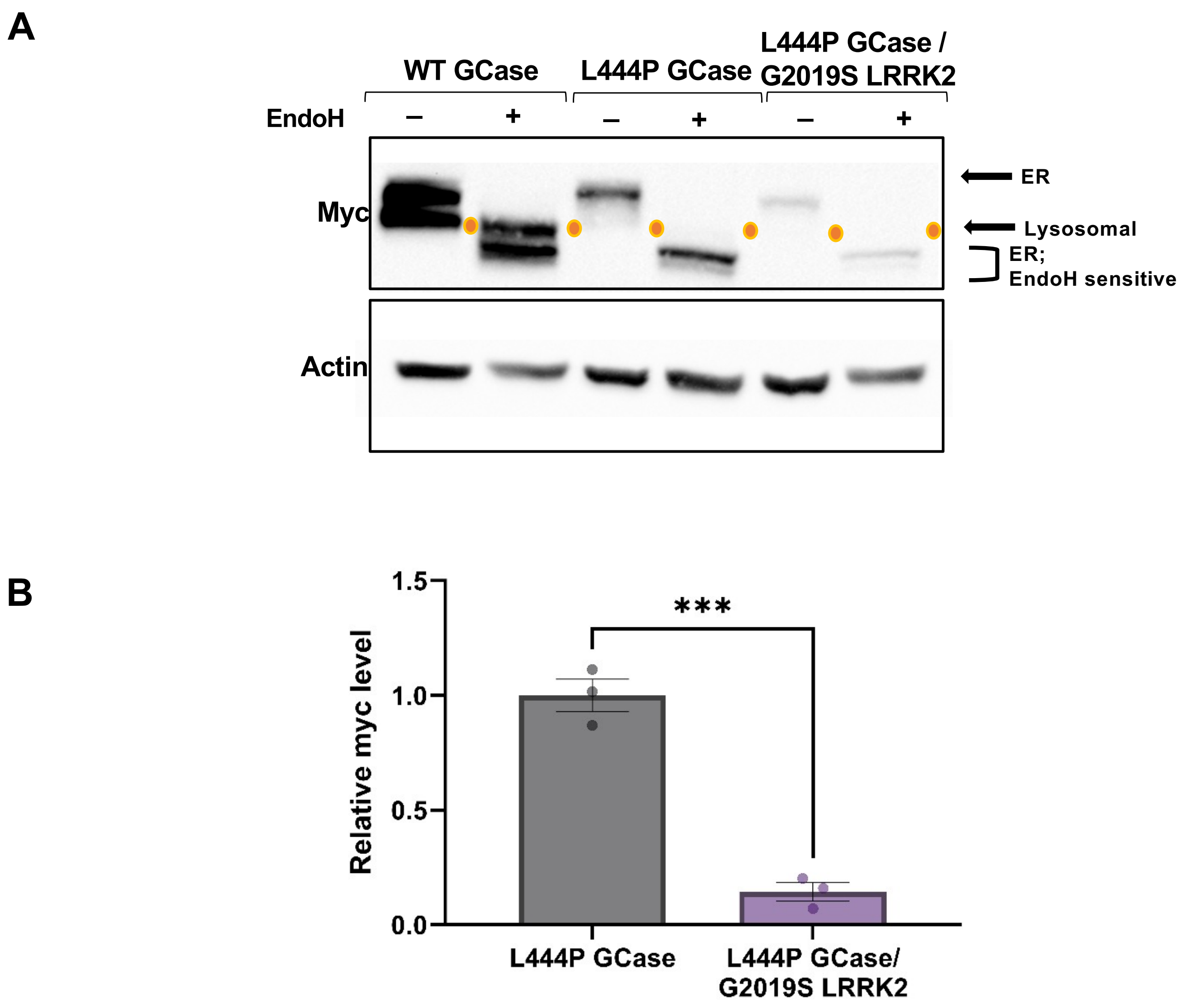

Figure S5. Steady state level of mutant GCase reduces in the presence of mutant LRRK2 in 22-days old flies. **A** Protein lysates were prepared from 10 flies expressing the human WT GCase, L444P GCase (grey bar), and both L444P GCase and G2019S LRRK2 (purple bar) at 22-days post-eclosion. Expression was under a Da-GAL4 driver. Samples were subjected to overnight endoH digestion, after which they were electrophoresed through SDS-PAGE and the corresponding blots were interacted with anti-myc antibody. As a loading control, the blots were interacted with anti-actin antibody. **B** Intensities of the corresponding bands in the untreated lanes as shown in (A) were quantified by densitometry the GCase amount was divided by that of actin at the same lane. The value obtained for untreated L444P GCase was considered 1 (paired student's t-test, p=0.0004). The results are the mean ± SEM of three independent experiments.

Supplementary Figure 6.

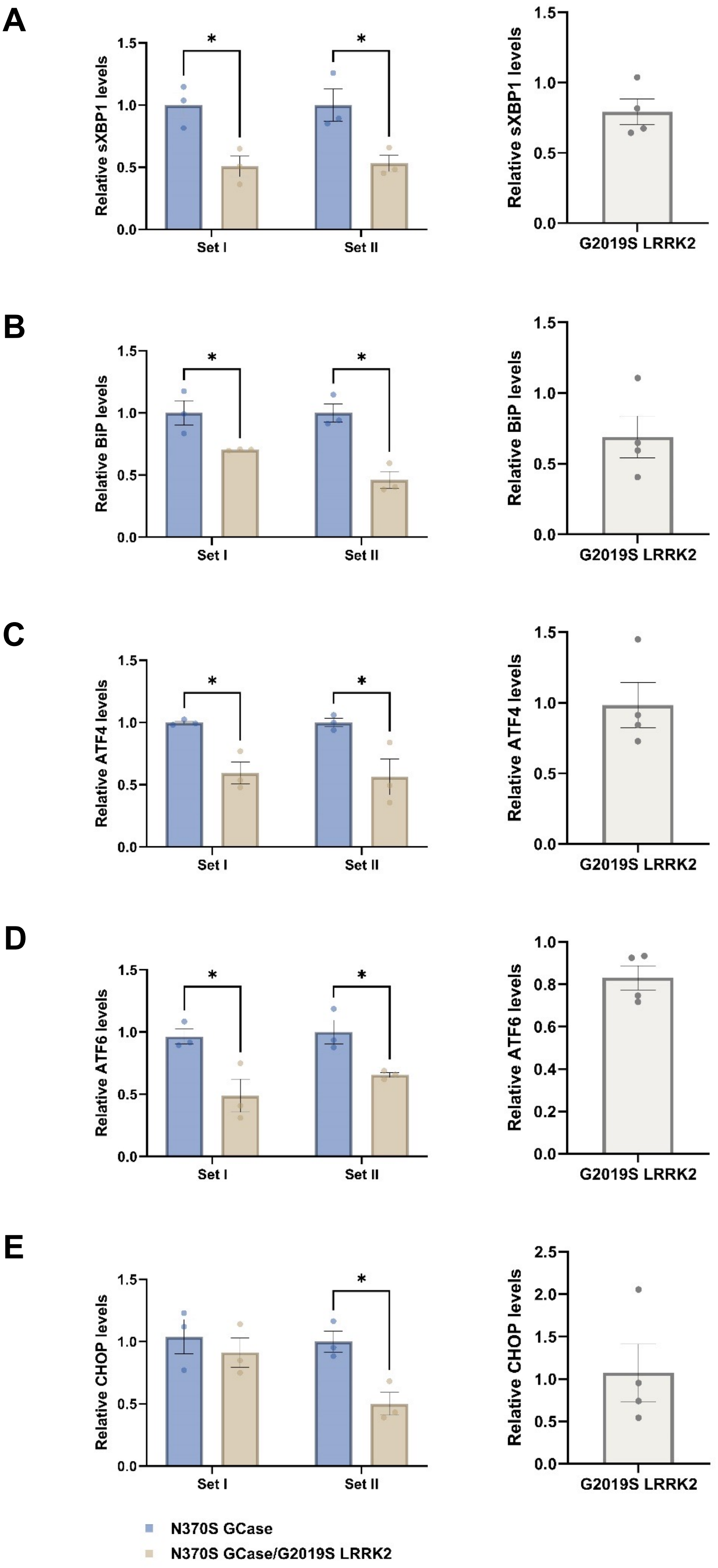

Figure S6. UPR in PD patients derived fibroblasts. mRNA levels of UPR markers (A) *sXBP1* (Set I p= 0.02, Set II p= 0.03), (B) *BiP* (Set I p= 0.04, Set II p= 0.006), (C) *ATF4* (Set I p= 0.01, Set II p= 0.04), (D) *ATF6* (Set I p= 0.03, Set II p= 0.02) and (E) *CHOP* (Set II p= 0.02) in PD patients-derived fibroblasts were analyzed by qRT-PCR. Paired student's t-test was used to calculate the significance of the result. GAPDH was used as a normalizing gene. The value obtained for the single mutant *GBA1* sample was considered 1. The results are the mean ± SEM of three independent experiments, performed on two sets of cells derived from one double mutant carrier and one *GBA1* carrier, grown in two different centers.

Supplementary Figure 7.

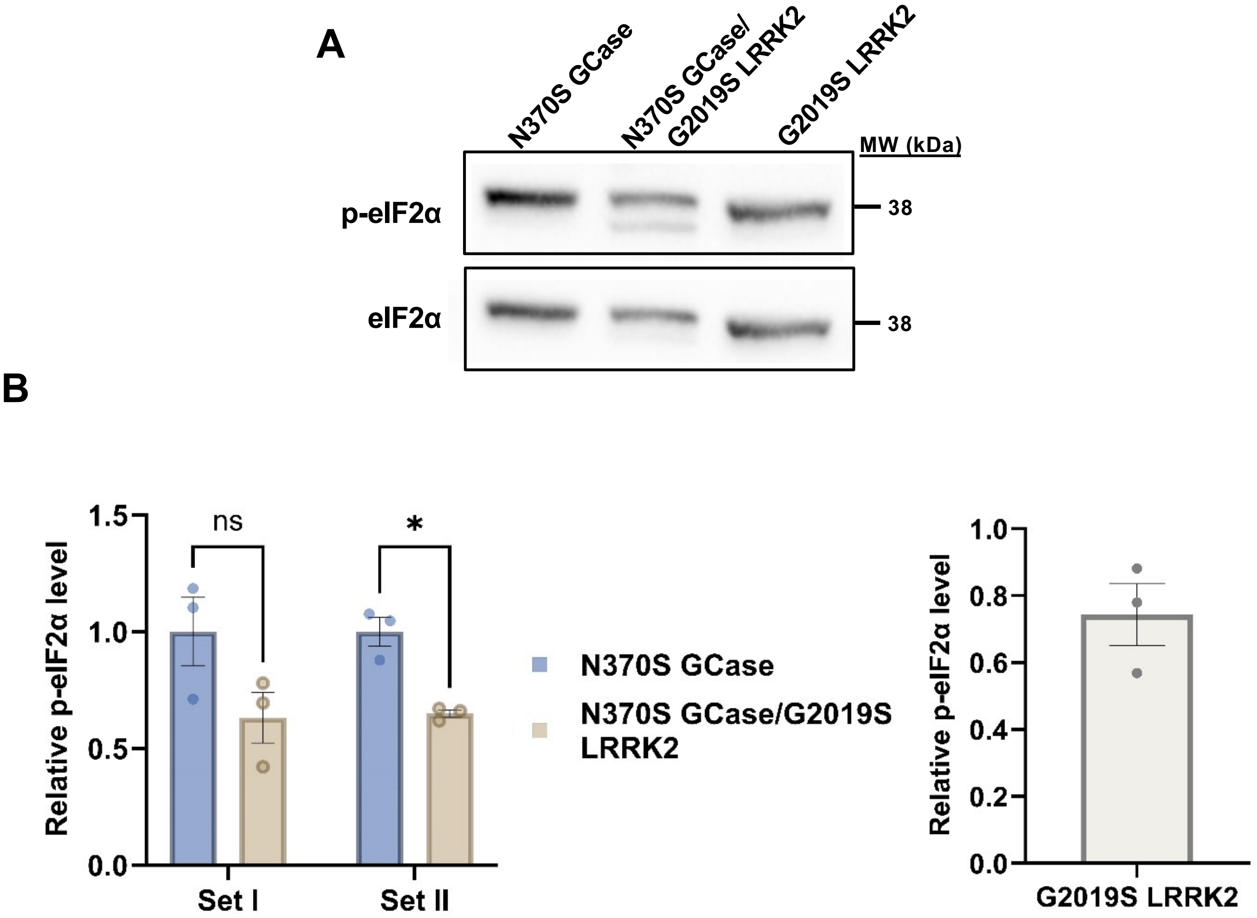

Figure S7. Phosphorylation of eIF2α in PD patient derived fibroblasts. A Protein lysates were prepared from PD patients' derived fibroblasts, carrying either the N370S *GBA1* mutation (blue bars), the G2019S LRRK2 mutation (white bar), or both N370S *GBA1* and G2019S *LRRK2* mutations (brown bars), and subjected to western blotting. The corresponding blots were interacted with anti-phospho-eIF2α antibodies. As a loading control, the blots were interacted with anti-eIF2α antibodies. **B** Intensities of the corresponding bands as shown in (A) were quantified by densitometry and the value obtained for single mutant *GBA1* sample was considered 1 (paired student's t-test, Set II p=0.005375) . The results are the mean ± SEM of three independent experiments.
